# Physiologically based pharmacokinetic modeling to assess metabolic drug–drug interaction risks and inform the drug label for fedratinib

**DOI:** 10.1007/s00280-020-04131-y

**Published:** 2020-09-04

**Authors:** Fan Wu, Gopal Krishna, Sekhar Surapaneni

**Affiliations:** 1 Nonclinical Research and Development, Bristol Myers Squibb, Summit, NJ USA; 2 Clinical Pharmacology, Bristol Myers Squibb, Summit, NJ USA

**Keywords:** Drug–drug interaction, PBPK, Metabolic inhibition, Simcyp, Fedratinib

## Abstract

**Purpose:**

Fedratinib (INREBIC^®^), a Janus kinase 2 inhibitor, is approved in the United States to treat patients with myelofibrosis. Fedratinib is not only a substrate of cytochrome P450 (CYP) enzymes, but also exhibits complex auto-inhibition, time-dependent inhibition, or mixed inhibition/induction of CYP enzymes including CYP3A. Therefore, a mechanistic modeling approach was used to characterize pharmacokinetic (PK) properties and assess drug–drug interaction (DDI) potentials for fedratinib under clinical scenarios.

**Methods:**

The physiologically based pharmacokinetic (PBPK) model of fedratinib was constructed in Simcyp^®^ (V17R1) by integrating available in vitro and in vivo information and was further parameterized and validated by using clinical PK data.

**Results:**

The validated PBPK model was applied to predict DDIs between fedratinib and CYP modulators or substrates. The model simulations indicated that the fedratinib-as-victim DDI extent in terms of geometric mean area under curve (AUC) at steady state is about twofold or 1.2-fold when strong or moderate CYP3A4 inhibitors, respectively, are co-administered with repeated doses of fedratinib. In addition, the PBPK model successfully captured the perpetrator DDI effect of fedratinib on a sensitive CY3A4 substrate midazolam and predicted minor effects of fedratinib on CYP2C8/9 substrates.

**Conclusions:**

The PBPK-DDI model of fedratinib facilitated drug development by identifying DDI potential, optimizing clinical study designs, supporting waivers for clinical studies, and informing drug label claims. Fedratinib dose should be reduced to 200 mg QD when a strong CYP3A4 inhibitor is co-administered and then re-escalated to 400 mg in a stepwise manner as tolerated after the strong CYP3A4 inhibitor is discontinued.

**Electronic supplementary material:**

The online version of this article (10.1007/s00280-020-04131-y) contains supplementary material, which is available to authorized users.

## Introduction

Fedratinib (INREBIC^®^, also known as SAR302503 or TG101348) is an oral, small molecule, selective Janus kinase 2 (JAK2) inhibitor, with activity against both wild-type and mutant JAK2, approved in the United States for the treatment of adult patients with myelofibrosis (MF) [1]. The approved dose of fedratinib is 400 mg once daily (QD). Fedratinib PK is characterized by rapid oral absorption, approximately dose-proportional PK over a dose range of 300–500 mg QD, high protein binding, metabolism primarily by cytochrome P450 (CYP) enzyme CYP3A and secondarily by CYP2C19, excretion via fecal route, and a long-terminal half-life [2]. In vitro studies reveal that fedratinib is not only a substrate of CYP3A and CYP2C19, but also exhibits complex auto-inhibition, time-dependent inhibition, or mixed inhibition/induction on CYP enzymes including CYP3A and CYP2C19 [2]. Therefore, it is crucial to use a mechanistic modeling approach to characterize pharmacokinetic (PK) properties and predict drug-drug interaction potentials for fedratinib under clinical scenarios.

Over the past decades, PBPK modeling has received rapidly increasing attention from academia, industry, and regulatory agencies [3–6]. In this modeling study, a PBPK model for fedratinib was first developed based on in vitro and in vivo data within Simcyp^®^ (Certara, Sheffield, UK), an extensively qualified and verified commercial PBPK software platform. The developed PBPK model was then verified or refined based on additional clinical pharmacokinetic (PK) and drug-drug interaction (DDI) observations for fedratinib. The verified PBPK models were applied to predict DDI between fedratinib and CYP modulators or CYP substrates. Further sensitivity analyses were conducted to identify key parameters impacting model predicted DDI effects. The PBPK model predictions were applied to inform drug label claims and guide dose adjustments under various clinical scenarios.

## Materials and methods

### Preclinical and clinical study data

Preclinical and clinical studies used to support the model development are summarized in Supplemental Material SM1. The clinical studies were conducted in accordance with the Declaration of Helsinki and the International Council for Harmonisation Guideline for Good Clinical Practice (ICH E6) and approved by ethics committees (see details in Supplemental Material SM1).

### Modeling strategy

The overall modeling strategy and procedure are described below with drug properties and model input parameters described in Supplemental Material SM2.

#### Overall modeling procedure

**Fig. 1:**
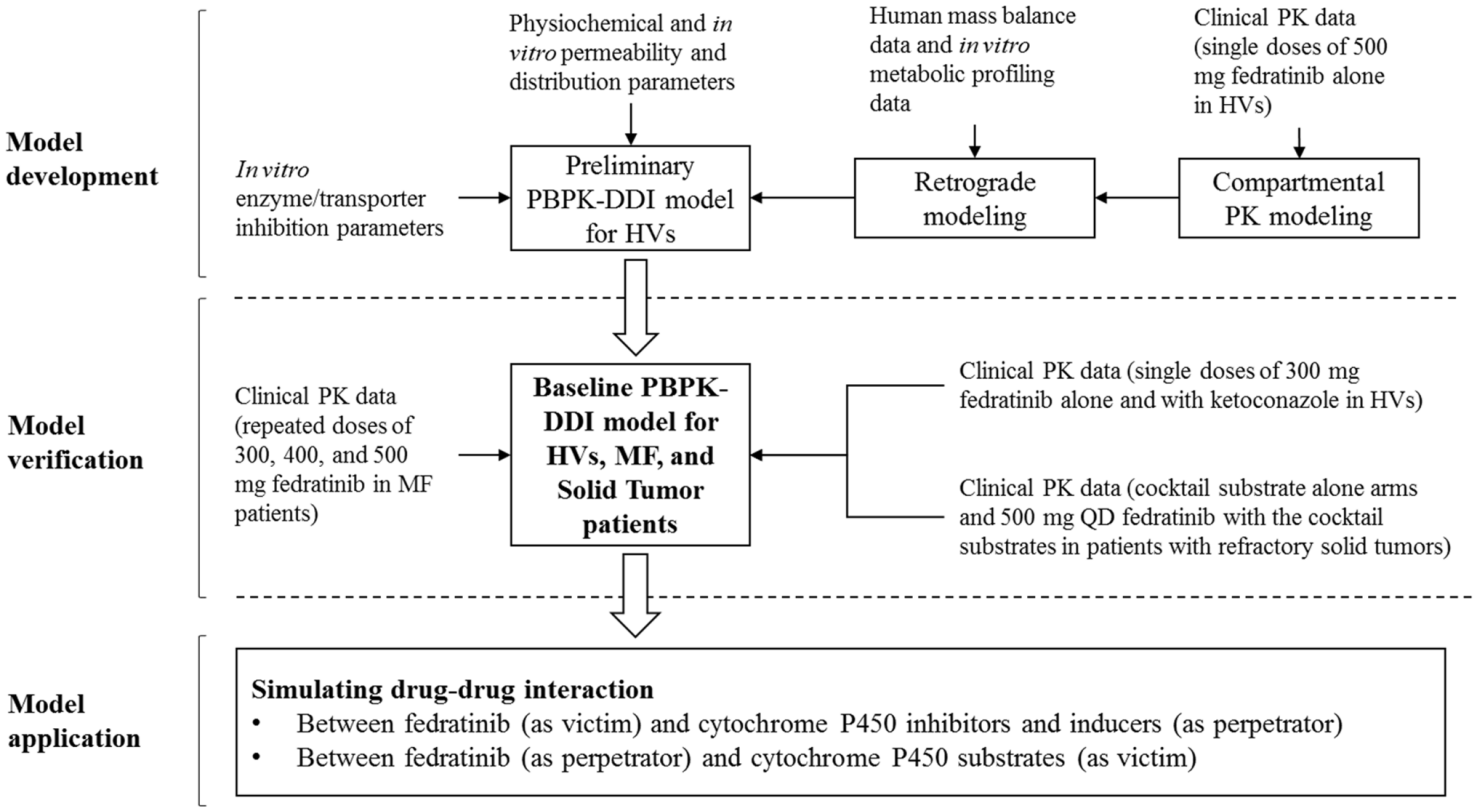
Modeling Procedures for the PBPK Model of Fedratinib

The modeling strategy for the PBPK model of fedratinib including the model development, model verification/modification, and model application is presented in Fig. 1, following recent regulatory guidance for industry [7].

#### Model development

The PBPK model of fedratinib was constructed in Simcyp^®^ by integrating available in vitro and in vivo information, including physiochemical parameters, permeability and blood binding parameters, enzyme and transporter inhibition parameters, and human PK parameters estimated from clinical PK data. A stepwise “middle-out” approach was applied to estimate the human PBPK parameters for fedratinib in Simcyp^®^. First, the temporal profiles of mean plasma concentration of fedratinib were collected from the clinical studies in healthy volunteers (HVs) administered single doses of 500 mg fedratinib. Then the preliminary human oral PK parameters were estimated as described in Supplemental Materials SM2 and SM3.

Using the retrograde modeling tool provided within Simcyp^®^, the intrinsic enzyme clearance (CL_int_) values were optimized by minimizing the differences between the model-predicted and clinically observed dose-normalized AUC_inf_ (see Supplemental Material SM2—Metabolism Parameters), with the fractions of metabolism of CYP3A4 (fm_CYP3A4_) and additional hepatic enzymes (fm_HLM,add_) refined by the parameter sensitivity analysis on accumulation ratio for repeated-dose clinical studies (see Supplemental Material SM8). The renal clearance (CL_R_) and additional systemic clearance (CL_add_) were provided as input parameters estimated from the clinical data (see Supplemental Material SM2—Excretion Parameters). The PBPK distribution parameters (including Vss, Vsac, and Q) were directly derived from the compartmental modeling of plasma PK data obtained from healthy subjects administered 500 mg fedratinib single doses, and then provided as input parameters without further modifications (see Supplemental Material SM2—Distribution Parameters).

#### Model verification and modification

First, the developed PBPK model was verified against clinical observations in healthy subjects administered single doses of 300 mg fedratinib alone without further modifications. Second, the developed PBPK model was verified against clinical data under repeated dosing scenarios. As no repeated-dosing studies have been conducted in healthy volunteers, the model verification was conducted using the clinical PK profiles from myelofibrosis (MF) patients administered repeated doses of 300, 400, or 500 mg fedratinib once a day (QD) [8]. Third, the PBPK model of fedratinib was verified against the clinical data observed in healthy volunteers co-dosed with fedratinib (300 mg, single dose) and ketoconazole (200 mg BID) [9]. Finally, the PBPK model was verified against the clinical observations in solid tumor patients co-administered fedratinib (500 mg QD) with CYP cocktail substrates (single doses of 2 mg midazolam, 20 mg omeprazole, and 100 mg metoprolol) [2].

#### Model application

Without further modification, the verified PBPK DDI model was applied to (1) predict DDI effects between fedratinib (as the victim) and CYP inhibitors and inducers (see Supplemental Material SM9 Table 7); (2) predict DDI effects between fedratinib (as the perpetrator) and CYP substrates, including repaglinide (CYP2C8 substrate) and warfarin (CYP2C9 substrate). The extent of the DDI was defined as the ratio of the geometric mean AUC from the co-administration to that from the alone treatment arm.

#### Parameter sensitivity analysis and prediction error

The parameter sensitivity analyses (PSA) were conducted using the “Sensitivity” Toolbox provided in Simcyp^®^ (V17R1) to investigate effects of key parameters on model predictions. All parameters of investigation were varied over a greater than tenfold range.

The prediction error (PE) is calculated using PE = (GM_prediction_ − GM_observation_)/GM_observation_ × 100%, where GM refers to geometric mean, and SD refers to standard deviation.

#### Modeling software and simulation design

The commercial population-based PBPK software Simcyp^®^ was used for model building purposes. The default compound and population library files within Simcyp^®^ were used in this modeling work without further modification. A summary table of input parameters for the Simcyp^®^ PBPK model of fedratinib is provided in Supplemental Material SM4. Any additional mathematical or statistical calculations beyond those reported by Simcyp^®^ are explicitly described in the “Methods” or “Results” section.

The simulation design information for each modeling stage is summarized in “Supplemental Material” (SM5 Table 4). In all simulations, fedratinib and other compounds were administered via the oral route in the fasted state unless indicated differently. In the table, HV and Cancer refer to “Sim-Healthy Volunteers.lbrz” and “Sim-Cancer.lbrz” Simcyp^®^ population files used in the corresponding simulations, respectively. The demographic parameters and the dosage settings were adjusted to meet the actual clinical patient information. In case that the actual age ranges exceed those allowed in Simcyp, the minimum or maximum age value was used accordingly.

## Results

### Simulation of fedratinib single doses in healthy subjects

**Fig. 2:**
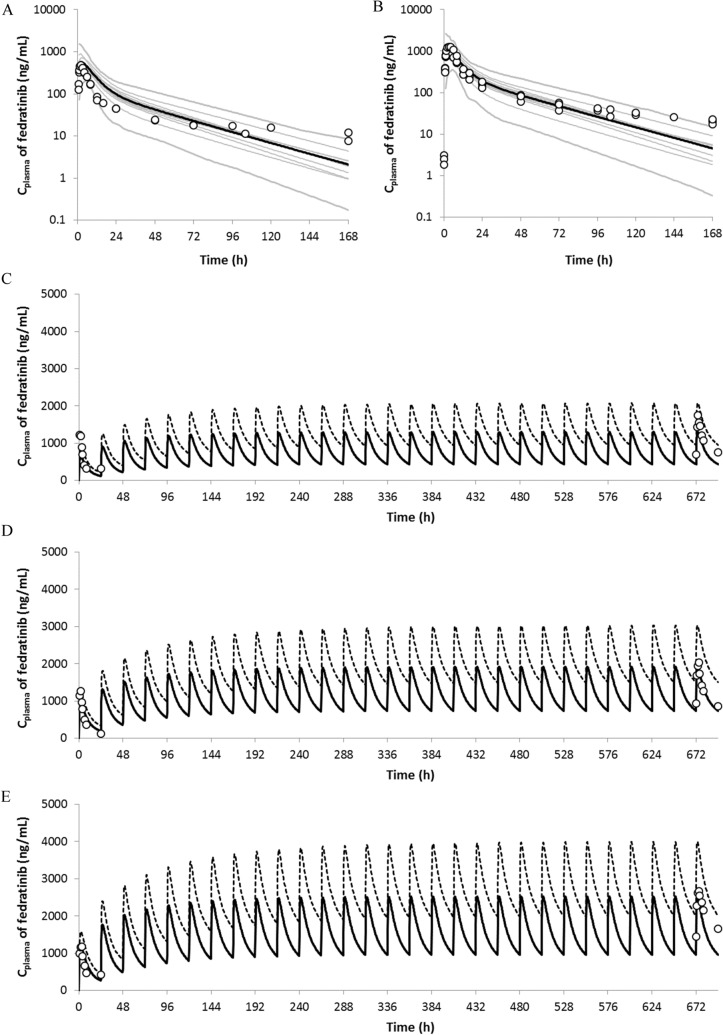
Model-simulated and Clinically Observed Mean Plasma Concentration Profiles of Fedratinib in Healthy Subjects Following Single Doses of 300 mg (Subplot **a**) or 500 mg (Subplot **b**) Fedratinib and in Myelofibrosis (MF) Patients Following Repeated Doses (QD) of 300 mg (Subplot **c**), 400 mg (Subplot **d**), and 500 mg (Subplot **e**) Fedratinib. The clinically observed mean plasma concentrations are represented as the open symbols. The mean and individual trial simulations using the default Simcyp^®^ “Healthy Volunteers” are represented as solid black and gray curves (Number of virtual trials = 10), respectively, in Subplot A and B. The mean simulations using the default Simcyp^®^ “Healthy Volunteers” and “Cancer” populations are presented and solid and dashed black curves in Subplot **c**–**e**

The PBPK model of fedratinib was developed by integrating both in vitro and in vivo information as described in the “Materials and Methods” section. Following the simulation design consistent with the clinical study protocols (see Supplemental Material SM5 Table 4), the simulations were conducted within Simcyp^®^ for healthy subjects given a single dose of 300 mg or 500 mg fedratinib. Overall, the model captures the clinically observed mean plasma concentration–time data, while under-prediction is found at the terminal stage with time later than ~ 96 h (see Fig. 2a, b). The corresponding model-simulated PK parameters agree with the clinical observations as summarized in Supplemental Material (SM 6 Table 5), while the prediction errors are 11% in Cmax and 31% in AUC_inf_ for 300 mg single-dose scenario and 39% in Cmax and 22% in AUC_inf_ for 500 mg single-dose scenario, respectively, implying consistent over-prediction of both Cmax and AUC_inf_ in comparison with the clinical single-dose PK data.

### Simulation of fedratinib multiple doses in myelofibrosis (MF) patients

Since no repeated-dose studies were conducted for fedratinib in healthy subjects, the model verifications were carried out based on the clinical studies conducted in MF patients following repeated doses of 300 mg, 400 mg, and 500 mg fedratinib. The simulations were conducted within Simcyp^®^ following the simulation design consistent with the clinical study protocol in which full PK profiles were obtained at Cycle 1 Day 1 and Cycle 2 Day 1 in consecutive 28-day cycles (see Supplemental Material SM5 Table 4).

The input model parameters used for the repeated dose simulations were kept the same as those used for the single dose simulations using either Simcyp “Healthy volunteers” or “Cancer” population files. As shown in Fig. 2c–e and Supplemental Material SM6 Table 5, the model-predicted and clinically observed concentration profiles and exposure parameters are aligned following repeated doses (QD) of 300 mg, 400 mg, and 500 mg fedratinib in MF patients, implying that the net effects of fedratinib-induced auto-inhibition and auto-induction on CYP3A4 are sufficiently captured by the current PBPK DDI model. Using the “Healthy volunteers” population file, the PBPK model prediction errors for steady-state exposure parameters are − 19% in Cmax and − 29% in AUCτ for 300 mg QD, 0% in Cmax and − 10% in AUCτ for 400 mg QD, and − 4% in Cmax and  14% in AUCτ for 500 mg QD, respectively.

### Simulation of the drug interaction of fedratinib (as the Victim) with ketoconazole in healthy subjects

Ketoconazole was known to be a potent CYP3A4/5 inhibitor and was used in clinical DDI studies as a probe inhibitor until being recently replaced by alternative clinical CYP3A4/5 inhibitors [10, 11]. The simulations of drug–drug interaction between fedratinib and ketoconazole were conducted within Simcyp^®^ following the simulation design (see Supplemental Material SM5 Table 4) consistent with the clinical protocol of ketoconazole and fedratinib DDI [9].

**Fig. 3:**
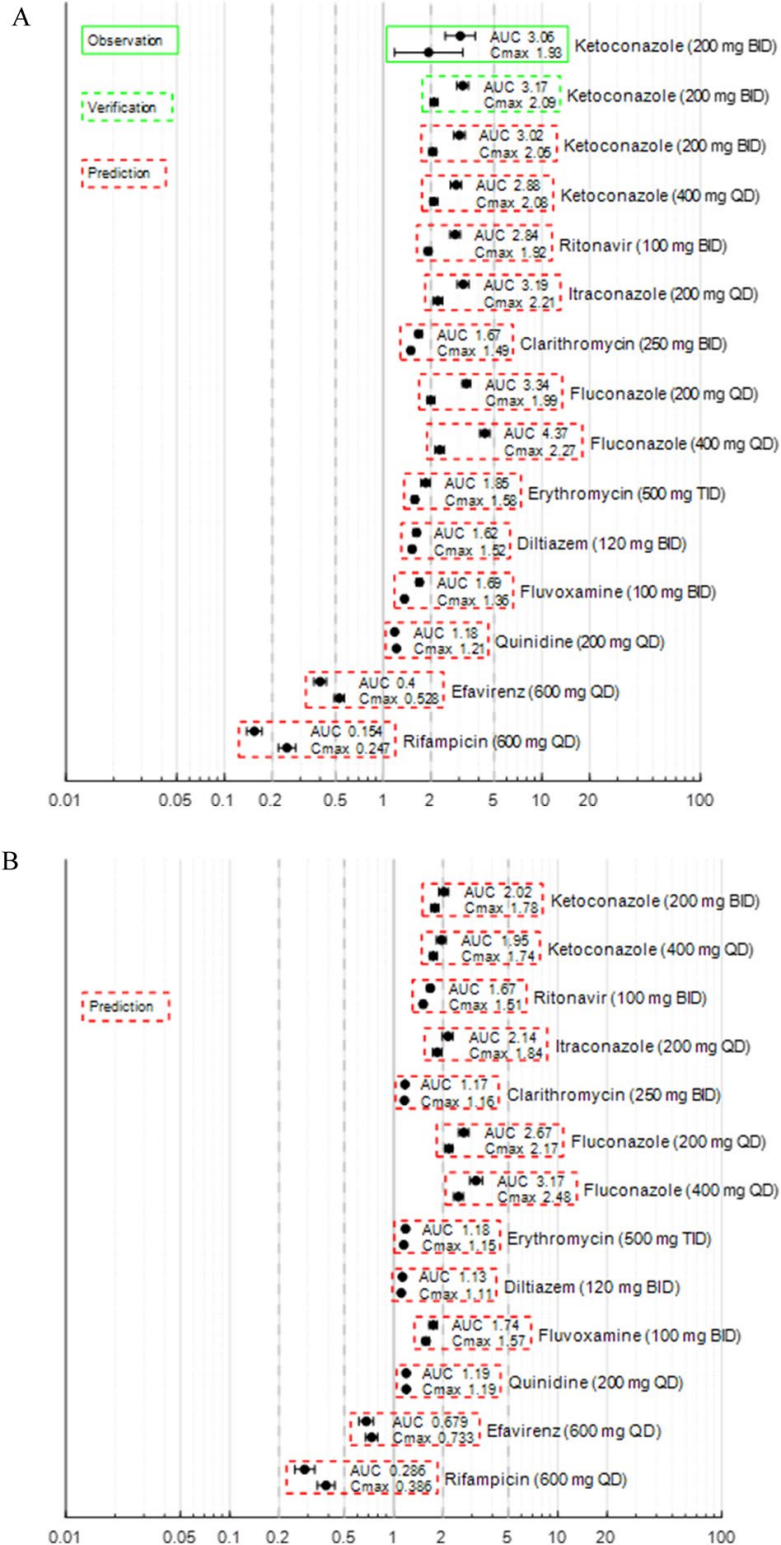
Summary of Model-predicted Drug-drug Interactions between Fedratinib (as the Victim) and CYP3A4 Modulators in Single Dose (Subplot **a**) and Repeated Dose (Subplot **b**) Scenarios. Subplot **a**: In the DDI simulations under the single-dose scenario, fedratinib was administered to healthy subjects as a single dose of either 300 mg (in the “Observation” and “Verification” categories) or 400 mg (in the “Prediction” category), and AUC refers to AUC_inf_. Subplot **b**: In the DDI simulations under the repeated-dose scenario, fedratinib was administered to healthy subjects as 400 mg QD (in the “Prediction” category), and AUC refers to AUC_τ_ at steady state. In both subplots, the symbols represent geometric means of the estimates and error bars represent the 90% confidence intervals of the estimates

The PBPK model was applied to simulate DDI between fedratinib and ketoconazole. As demonstrated by the simulated PK profiles (see Supplemental Material SM 7 Fig. 3a) and exposure parameters (e.g., simulated geometric mean AUC ratio = 3.17 versus observed AUC ratio = 3.06) (see Table 1), the PBPK model was found to sufficiently capture the clinically observed DDI between fedratinib and ketoconazole observed in healthy volunteers given a single dose of 300 mg fedratinib and repeated doses (BID) of 200 mg ketoconazole. The PBPK model prediction errors are 40% in Cmax and 32% in AUC_inf_ for fedratinib alone, and 50% in Cmax and 8% in AUC_inf_ for fedratinib co-dosed with ketoconazole, respectively. Further parameter analyses reveal that the model-predicted DDI between fedratinib and ketoconazole is sensitive to changes in metabolic contribution of CYP3A4 (or fm_CYP3A4_) of fedratinib (see Supplemental Material SM 8 Table 6). Overall, the simulations reveal that the current PBPK DDI model provides a reasonable estimate of fm_CYP3A4_ in vivo.

**Table 1:** Summary of model-simulated and clinically observed exposure parameters in DDI studies with fedratinib as the victim or perpetrator

Clinical scenario	Virtual subjects	Cmax (ng/mL)			AUC (ng/mL·h)		
		Observation	Prediction	PE (%)	Observation	Prediction	PE (%)
Fedratinib as the Victim: Fedratinib administered (300 mg single dose) with and without ketoconazole (CYP3A4 inhibitor, 200 mg BID) in healthy subjects							
FEDR alone Mean (SD) [GM]	Healthy	444 (262) [379]	645 (471) [529]	40	7440 (4060) [6460]	10,200 (6800) [8550]	32
FEDR + KTZ Mean (SD) [GM]	Healthy	812 (374) [741]	1390 (1030) [1110]	50	26,800 (10,600) [25000]	31,200 (15,700) [27100]	8
GM Ratio (90% CI)	Healthy	1.93 (1.18–3.17)	2.09 (1.98–2.21)	8	3.06 (2.46–3.80)	3.17 (2.91–3.45)	4
Fedratinib as the Perpetrator: Midazolam (CYP3A4 substrate, 2 mg single dose) administered as a cocktail with and without fedratinib (500 mg QD) in cancer patients							
MDZ alone Mean (SD) [GM]	Healthy	14.8 (7.56) [13.4]	8.91 (6.64) [7.27]	− 46	52.2 (37.9) [41.5]	30.9 (20.8) [24.5]	− 41
	Cancer		9.54 (6.67) [7.84]	− 41		34.3 (22.8) [27.0]	− 35
MDZ + FEDR Mean (SD) [GM]	Healthy	25.9 (8.75) [24.2]	18.4 (14.2) [14.7]	− 39	152 (38.6) [148]	181 (189) [104]	− 30
	Cancer		20.3 (14.2) [16.6]	− 31		222 (249) [130]	− 12
GM Ratio (90% CI)	Healthy	1.82 (1.49–2.21)	2.02 (1.95–2.10)	11	3.84 (2.62–5.63)	4.26 (3.87–4.69)	11
	Cancer		2.11 (2.03–2.20)	14		4.82 (4.35–5.35)	26
Fedratinib as the Perpetrator: Omeprazole (CYP2C19 substrate, 20 mg once) administered as a cocktail with and without fedratinib (500 mg QD) in cancer patients							
OMEP alone Mean (SD) [GM]	Healthy	550 (285) [475]	313 (218) [248]	− 48	2210 (1740) [1590]	764 (903) [510]	− 68
	Cancer		352 (223) [291]	− 39		879 (1250) [616]	− 61
OMEP + FEDR Mean (SD) [GM]	Healthy	615 (286) [537]	430 (325) [326]	− 39	5950 (3560) [4690]	1640 (4930) [743]	− 84
	Cancer		495 (325) [398]	− 26		1850 (4390) [947]	− 80
GM Ratio (90% CI)	Healthy	1.12 (0.81–1.53)	1.32 (1.29–1.34)	18	2.82 (2.26–3.53)	1.46 (1.40–1.51)	− 48
	Cancer		1.36 (1.34–1.39)	21		1.54 (1.47–1.61)	− 45
Fedratinib as the Perpetrator: Metoprolol (CYP2D6 substrate, 100 mg once) administered as a cocktail with and without fedratinib (500 mg QD) in cancer patients							
Metoprolol alone Mean (SD) [GM]	Healthy	155 (79.1) [135]	164 (77.5) [147]	9	880 (545) [731]	1510 (1890) [1060]	45
	Cancer		199 (87.3) [179]	33		2100 (2320) [1460]	100
Metoprolol + FEDR Mean (SD) [GM]	Healthy	221 (106) [199]	176 (86.2) [157]	− 21	1460 (938) [1180]	2150 (3870) [1220]	3
	Cancer		213 (95.5) [191]	− 4		2840 (4400) [1690]	43
GM Ratio (90% CI)	Healthy	1.60 (1.25–2.05)	1.07 (1.06–1.07)	− 33	1.77 (1.27–2.47)	1.15 (1.12–1.18)	− 35
	Cancer		1.07 (1.07–1.07)	− 33		1.16 (1.13–1.18)	− 34

AUC refers to AUC_inf_ for the single dose scenarios and AUCτ for the repeated QD dose scenarios. The number of subjects (*n*) = 6–7 in INT12893, *n* = 13–16 in INT12497, and *n* = 100 in virtual trial simulations, respectively. All model simulations listed in the table were simulated using the default “Healthy Volunteers” and “Cancer” population files provided in Simcyp^®^ (V17R1). The prediction error is calculated using PE = (GM_prediction_ − GM_observation_)/GM_observation_ × 100%, where GM refers to geometric mean. SD refers to standard deviation. *FEDR* fedratinib, *KTZ* ketoconazole, *MDZ* midazolam, *OMEP* omeprazole

### Simulation of the drug interaction of fedratinib (as the Perpetrator) with the cocktail substrates

**Fig. 4:**
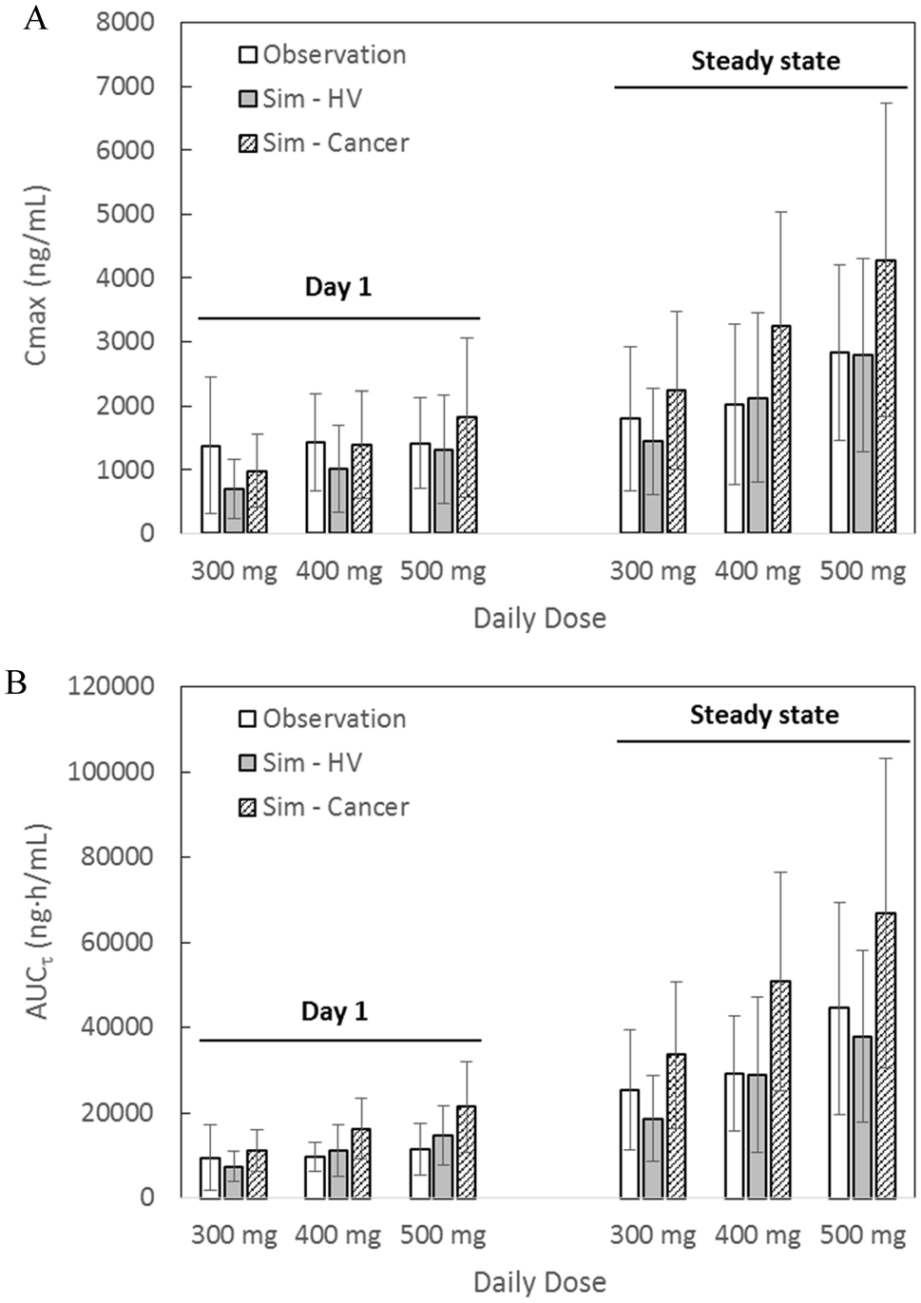
Comparison between Model-Predicted and Clinically Observed Cmax (Subplot **a**) and AUC_τ_ (Subplot **b**) in Myelofibrosis Patients

The simulations of drug–drug interaction between CYP cocktail substrates (including midazolam as CYP3A4 probe substrate [12], omeprazole as CYP2C19 probe substrate [13], and metoprolol as CYP2D6 probe substrate [14]) were conducted for patients with refractory solid tumors using both the default Simcyp^®^ “Healthy Volunteers” and “Cancer” population model files without any further modifications of the baseline model parameters. The model-simulated PK profiles and exposure parameters along with interaction magnitudes for the cocktail substrates are summarized in Supplemental Material SM 7 Fig. 4b–d and Table 1, respectively.

For midazolam, the simulations captured the interaction magnitude with the simulated geometric mean Cmax ratio = 2.02 (using the “Healthy Volunteers” population file) or 2.11 (using the “Cancer” population file) versus the observed 1.82, and the simulated AUC ratio = 4.26 (using the “Healthy Volunteers” population file) or 4.82 (using the “Cancer” population file) versus the observed 3.84. Using the “Healthy volunteers” population file, the PBPK model prediction errors for exposure parameters are − 46% in Cmax and − 41% in AUC_inf_ for midazolam alone, and − 39% in Cmax and − 30% in AUC_inf_ for midazolam co-dosed with fedratinib, respectively.

For omeprazole, the PBPK model underpredicted the AUC ratio (i.e., 1.46 and 1.54 using the “Healthy Volunteers” and “Cancer” population files, respectively, versus the observed 2.82) while it predicted similar Cmax ratio (i.e., 1.32 and 1.36 using the “Healthy Volunteers” and “Cancer” population files, respectively, versus the observed 1.12). Using the “Healthy volunteers” population file, the PBPK model prediction errors for exposure parameters are − 48% in Cmax and − 68% in AUC_inf_ for omeprazole alone, and − 39% in Cmax and − 84% in AUC_inf_ for omeprazole co-dosed with fedratinib, respectively.

For metoprolol, the PBPK model underpredicted both AUC ratio (i.e., 1.15 and 1.16 using the “Healthy Volunteers” and “Cancer” population files, respectively, versus the observed 1.77) and Cmax ratio (i.e., 1.07 using both “Healthy Volunteers” and “Cancer” population files versus the observed 1.60). Using the “Healthy volunteers” population file, the PBPK model prediction errors for exposure parameters are 9% in Cmax and 45% in AUC_inf_ for metoprolol alone, and − 21% in Cmax and 3% in AUC_inf_ for metoprolol co-dosed with fedratinib, respectively.

### Prediction of the drug interaction of fedratinib (as the Victim) with CYP modulators

The PBPK DDI simulations were conducted between single doses of fedratinib and repeated doses of CYP3A4 modulators in healthy subjects and cancer patients following the simulation design listed in Supplemental Material SM5. In the simulations, fedratinib was administered as a single dose (400 mg) or repeated doses (400 mg QD) with the modulators (as perpetrators) administered as repeated doses. The 400-mg dosing strength is consistent with the approved clinical efficacious dose [1, 2]. The class of each modulator along with victim and perpetrator dose regimen is summarized in Supplemental Material (SM9 Table 7).

The baseline PBPK model was applied to evaluate DDI between fedratinib (as the victim) and CYP modulators without any further modifications. The model-predicted drug exposure parameters and interaction magnitudes using the “Healthy Volunteers” and “Cancer” Simcyp^®^ population files are tabulated in Supplemental Material SM9. As a high-level summary of the DDI simulation findings, the model-predicted geometric mean AUC and Cmax ratios are summarized in forest plots, Fig. 3a, b, for single-dose and repeated-dose scenarios, respectively. The simulations and observations obtained for the clinical drug interaction studies between fedratinib and ketoconazole are also included in Fig. 3a for comparison. The DDI magnitudes were predicted to be lower in the “Cancer” population than those in the “Healthy Volunteers” population, and lower under the repeated-dose scenario than those under the single-dose scenario.

In summary, under the single-dose scenario, the extent of fedratinib-as-victim drug interactions in terms of geometric mean AUC ratio was predicted to be around threefold with strong CYP3A4 inhibitors, between 1.5- and twofold with moderate CYP3A4 inhibitors, around 1.2-fold with weak CYP3A4 inhibitors, around 0.4-fold with a moderate CYP3A4 inducer, and around 0.15-fold with a strong CYP3A4 inducer. The additional DDI simulation with fluconazole (a dual CYP2C19/CYP3A4 inhibitor) given 400 mg QD suggests a strong interaction with the AUC ratio of ~ 4.4-fold and the results are consistent with the contribution of CYP3A and CYP2C19 contribution to the overall metabolism of fedratinib. Compared with the single-dose scenario, the PBPK simulations predicted lower interaction magnitudes following repeated doses of fedratinib in the presence of the CYP modulators, i.e., AUC ratio of ~ twofold with strong CYP3A4 inhibitors, ~ 1.2-fold with moderate or weak CYP3A4 inhibitors, ~ threefold with a dual CYP2C19/CYP3A4 inhibitor, ~ 0.7-fold with a moderate CYP3A4 inducer, and ~ 0.3-fold with a strong CYP3A4 inducer.

### Prediction of the drug interaction of fedratinib (as the Perpetrator) with CYP substrates

The PBPK DDI simulations were conducted between repeated doses (400 mg QD) of fedratinib and single doses of probe substrates for CYP enzymes in healthy subjects (using the Simcyp “Healthy Volunteers” population file) and cancer patients (using the Simcyp “Cancer” population file) following the simulation design listed in Supplemental Material SM5. The probe substrates along with victim and perpetrator dose regimen are summarized in Table 2.

**Table 2:** Summary of model-simulated exposure parameters in DDI studies with fedratinib as the perpetrator on CYP2C8 or CYP2C9

Clinical scenario	Cmax (ng/mL)		AUC (ng/mL·h)	
	Prediction—healthy	Prediction—cancer	Prediction—healthy	Prediction—cancer
Fedratinib as the Perpetrator: Repaglinide (CYP2C8 substrate, 0.25 mg single dose) administered with and without fedratinib (400 mg QD)				
Repaglinide alone	3.83 (1.52) [3.51]	4.09 (1.50) [3.80]	9.01 (5.20) [7.53]	10.1 (6.19) [8.48]
Repaglinide + FEDR	4.92 (1.96) [4.49]	5.29 (1.98) [4.90]	13.8 (9.0) [11.0]	15.4 (10.6) [12.4]
GM Ratio (90% CI)	1.28 (1.25–1.30)	1.29 (1.26–1.31)	1.46 (1.41–1.51)	1.46 (1.41–1.51)
Fedratinib as the Perpetrator: Warfarin (CYP2C9 substrate, 15 mg single dose) administered with and without fedratinib (400 mg QD)				
Warfarin alone	1380 (466) [1310]	1540 (499) [1460]	68,000 (52,300) [52,000]	96,700 (100,000) [71,200]
Warfarin + FEDR	1380 (467) [1310]	1540 (500) [1460]	68,300 (52,500) [52,200]	97,200 (101,000) [71,600]
GM Ratio (90% CI)	1.00 (1.00–1.00)	1.00 (1.00–1.00)	1.00 (1.00–1.00)	1.01 (1.00–1.01)

AUC refers to AUC_inf_ for the single dose scenarios. The number of subjects *n* = 100 in virtual trial simulations, respectively. All model simulations listed in the table were simulated using the default “Healthy Volunteers” and “Cancer” populations provided in Simcyp^®^ (V17R1). *FEDR* fedratinib, *MDZ* midazolam

In healthy subjects, the extent of drug interactions in terms of geometric mean AUC and Cmax ratios were predicted to be approximately 1.5-fold and 1.3-fold with a CYP2C8 probe substrate (repaglinide), respectively; 1.0-fold and 1.0-fold with a CYP2C9 probe substrate (warfarin), respectively (see Table 2). The simulations suggested that the interaction magnitudes between fedratinib and the probe substrates in cancer patients were similar to those predicted in healthy subjects.

## Discussion

### Mechanistic modeling to capture clinical PK of fedratinib

The PBPK model of fedratinib was able to capture clinical PK profiles of fedratinib under both single-dose and repeated-dose scenarios in both healthy subjects and MF patients (see Fig. 2), although a further refinement of the distribution parameters can improve the model fittings at the terminal stage of the single-dose PK profiles in healthy subjects (see Supplemental Material SM12). A comparison between model-predicted and clinically observed exposure parameters in MF patients is shown in Fig. 4a, b. In the PBPK simulations, either the “Healthy Volunteers” or “Cancer” population file (provided in Simcyp^®^, V17R1) was applied with necessary adjustment in demographic parameters to match the reported patient values. The comparison shows that, when the “Healthy Volunteers” population was applied, model prediction errors of AUC and Cmax stay within ~ 1.5 fold for both single-dose and repeated-dose scenarios (see Supplemental Material SM 6 Table 5). Being consistent with the clinical observation [2], the model predicted an approximately dose-proportional increase in steady-state AUC and Cmax as doses increase from 300 mg QD to 400 mg QD. Besides, the model predicted accumulation ratios of ~ 2.2 and ~ 2.5 (Cmax and AUC, respectively) using the “Healthy Volunteers” population file, and ~ 2.4 and ~ 3.0 (Cmax and AUC, respectively) using the “Cancer” population profiles, and, in agreement with the clinical observation (i.e., accumulation ratios of 1.4–2.0 in Cmax and 2.9–3.2 in AUC, respectively) in MF patients (see SM 6 Table 5 and [15]).

Although fedratinib was determined to be a P‐gp substrate in vitro, fedratinib showed rapid absorption in clinical studies with high bioavailability [15], indicating that permeability and efflux are not limiting its absorption in clinically relevant dose range of 300 to 500 mg (see “Absorption Parameters” in Supplemental Material SM2). In addition, the patients with polycythemia vera (PV) are characterized by an abnormal increase in hemoglobin or hematocrit [16]. The potential effects of pathological changes in hematocrit on patient PK are not considered in the current PBPK model since the hematocrit values stayed within the normal range during the screening and treatment periods in patients. The changes in hematocrit may need to be considered if the data show changes in hematocrit upon treatment with fedratinib.

### Adequate modeling assessment on DDI magnitudes between fedratinib and CYP3A4 inhibitors or substrates

Polypharmacy is common in MF Patients [17, 18]. It is thus important to assess DDI risks from changed systemic drug exposure. The CYPs are the most abundant human enzymes that metabolize numerous xenobiotics including drugs, and as a result contribute to many DDIs. Since the in vitro metabolic profile data suggested that fedratinib is metabolized by multiple CYPs, with a predominant contribution from CYP3A4, as per the regulatory guidance [19–21], the clinical DDI study using a strong CYP3A4 inhibitor, ketoconazole, as well as the PBPK DDI modeling was conducted to provide a full mechanistic assessment of clinical DDI potentials. As shown in Table 1, the model-predicted DDI magnitudes overall match the clinically observed ketoconazole effects on fedratinib PK in healthy subjects, with prediction errors of less than 10%. In addition, the predicted geometric mean PK parameters including Cmax and AUC for fedratinib are also within 50% of the observed values. The simulation results indicate that the fraction of metabolism mediated by CYP3A4 (fm_CYP3A4_) of fedratinib given as a single dose is reasonably captured in the current PBPK model.

**Fig. 5:**
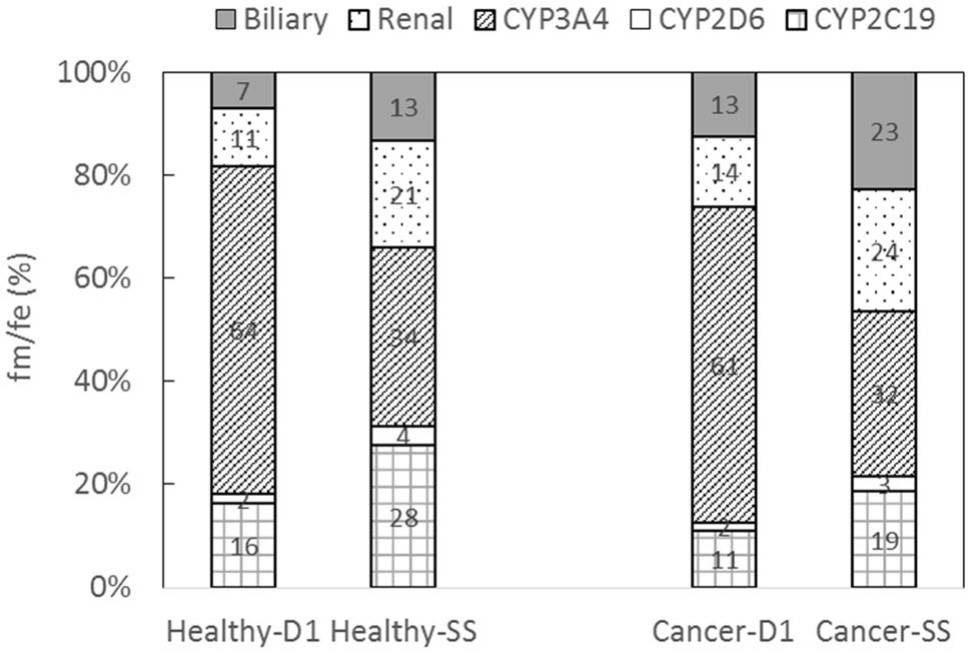
Model-simulated Fractions of Metabolism or Excretion (Fm/Fe) in Healthy Subjects and MF patients Following Repeated Doses of 400 mg QD Fedratinib. *D1* Day 1, *SS* Steady state

The in vitro studies also suggested that fedratinib inhibits and induces CYP3A4 activities in a time-dependent manner. By incorporating the auto-inhibition and auto-induction parameters, the PBPK model was also able to capture PK profiles following repeated doses of fedratinib (300–500 mg QD) in MF patients using the default “Healthy Volunteers” population file (see Supplemental Material SM6). In healthy subjects, the mean fractions of metabolism (fm) or excretion (fe) in relation to systemic clearance were estimated to be 16% for CYP2C19 (liver), 2% for CYP2D6 (liver), 64% for CYP3A4 (liver), 11% for the renal pathway, and 7% for the biliary pathway (see Fig. 5) following the first dose of 400 mg QD fedratinib, which are overall in good agreement with the in vitro observation. As measured from the cryopreserved human hepatocytes incubated with 1 µM fedratinib, the metabolic contributions to the hepatic clearance are ~ 17% for CYP2C19 (liver), ~ 2% for CYP2D6 (liver), ~ 63% for CYP3A (liver), and ~ 18% for the other liver enzymes, respectively. Following repeated doses, as a net effect of time-dependent inhibition and induction, fm of CYP3A4 was predicted to decrease to ~ 34%, while fm of CYP2C19 increased to ~ 28%, fm of CYP2D6 increased to ~ 4%, fe of the renal pathway increased to ~ 21%, and fe of biliary pathway increased to ~ 13% in healthy subjects (see Fig. 5). The model simulations also suggest the similar trends of time-dependent fm/fe changes in cancer patients (see Fig. 5). The time-dependent fm or fe provides mechanistic bases for the model-predicted attenuated metabolic DDI effects of CYP modulators on fedratinib under the repeated-dose scenario (see Fig. 3b).

The time-dependent fm_CYP3A4_ captured by the current PBPK model was further verified by the agreement between model-predicted and clinically observed fedratinib perpetrator effects on the index CYP3A4 substrate midazolam. According to the clinical observations, the AUC and Cmax of midazolam increased about fourfold and twofold, respectively, following repeated doses (500 mg QD) of fedratinib. The model-predicted AUC and Cmax ratios matched the clinical observations with prediction errors around 10%.

Therefore, the PBPK model is expected to adequately assess DDI potentials between CYP3A4 inhibitors and fedratinib under the single-dose and repeated-dose scenarios (see Fig. 3). Additionally, the PBPK model is expected to predict the perpetrator effect of fedratinib on a sensitive CYP3A4 substrate.

### Adequate modeling assessment on DDI magnitudes between fedratinib (as the perpetrator) and CYP2C8 or CYP2C9 substrates

The PBPK DDI simulations were conducted using the fedratinib PBPK model described here and the default repaglinide and warfarin compound models provided in Simcyp^®^ (V17R1). As summarized in Table 2, the model-predicted DDI effects are less than 50% for repaglinide (CYP2C8 substrate) and negligible for warfarin (CYP2C9 substrate), respectively, between repeated doses of fedratinib (400 mg QD) and single doses of repaglinide or warfarin (see Supplemental Material SM5 for the simulation designs). The development and verification of the Simcyp repaglinide and warfarin compound models were described elsewhere [22, 23]. For repaglinide, the parameter sensitivity analyses (PSAs) reveal that more than 20-fold changes in fedratinib Ki values for CYP2C8, CYP3A4, and OATP1B1 resulted in minor impacts on the predicted DDI magnitudes in terms of the repaglinide AUC ratio (see Supplemental Material SM 8). For warfarin, the PSAs reveal minor impacts of fedratinib Ki value for CYP2C9 on the warfarin AUC ratio. In summary, the PBPK simulations suggest insignificant perpetrator effects of fedratinib on repaglinide and warfarin PK under clinical scenarios.

### Model simulations to inform the drug label

**Fig. 6:**
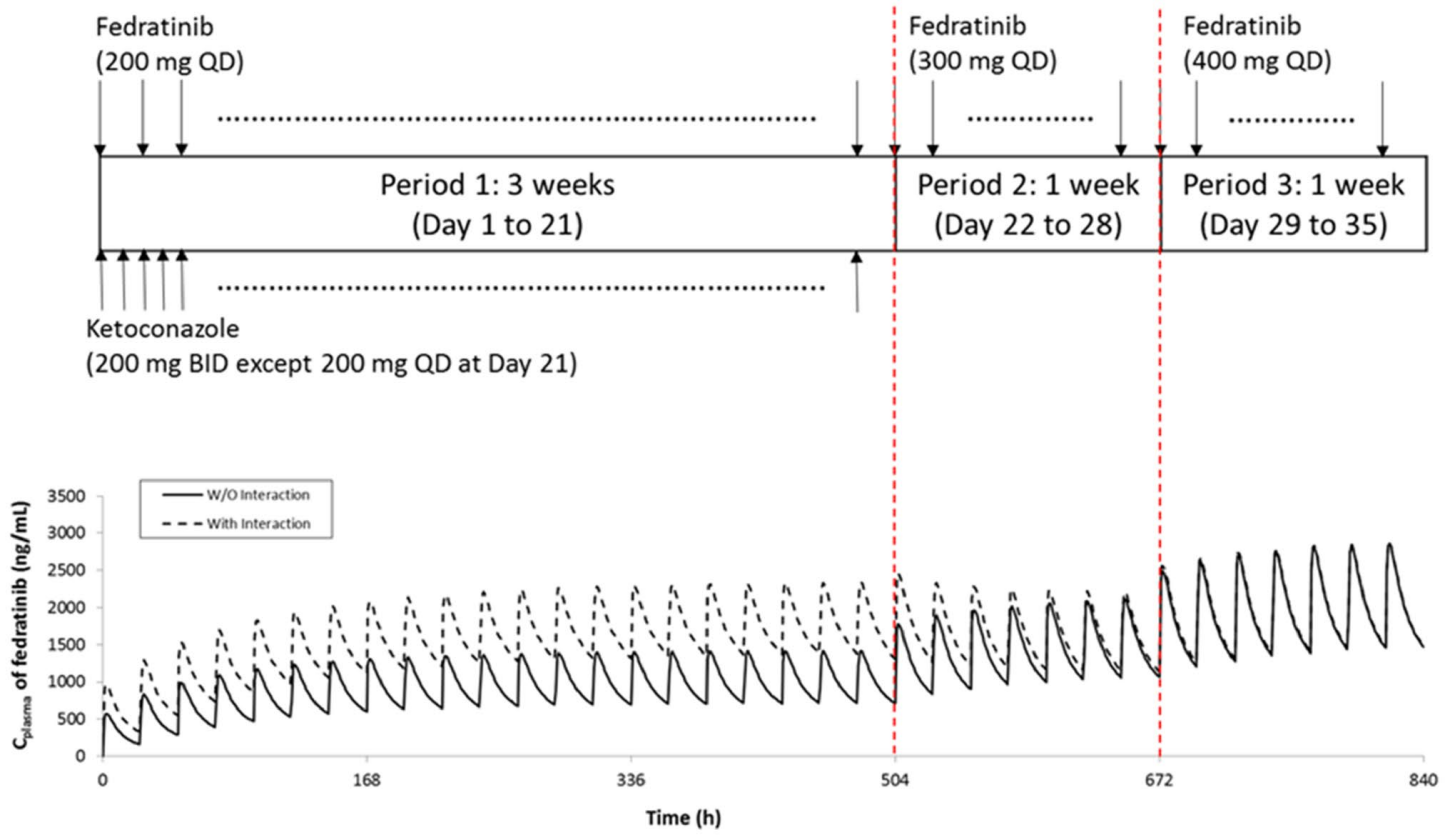
PBPK Simulation Design for Fedratinib Dose Re-Escalation after Discontinuation of Ketoconazole and the Simulated PK Profiles of Fedratinib in Cancer Patients

The PBPK simulations were applied to inform the drug label for fedratinib [1]. Based on the model-predicted DDI potential at steady state following repeated doses (400 mg QD) of fedratinib (Fig. 3), the drug label recommends to reduce the fedratinib dose to 200 mg QD when strong CYP3A4 inhibitors (such as ketoconazole and itraconazole) are co-administered with fedratinib. When mild or moderate CYP3A4 inhibitors are co-administered (such as diltiazem and erythromycin), it is recommended to maintain fedratinib dose at 400 mg QD as the increases in AUC and Cmax are predicted to be less than 20% at steady state (Fig. 3). Further, as supported by additional PBPK simulations (see Fig. 6 and Supplemental Material SM10), in cases where co-administration with a strong CYP3A4 inhibitor is discontinued, fedratinib dosage should be increased to 300 mg once daily during the first two weeks after discontinuation of the CYP3A4 inhibitor, and then to 400 mg once daily thereafter as tolerated.

### Current model limitations and future improvement

As discussed previously, the current PBPK model is adequate to evaluate DDI between fedratinib and CYP3A4 inhibitors, between fedratinib and CYP3A4 sensitive substrates, and between fedratinib and CYP2C8 or CYP2C9 substrates. However, the current model may not be applicable to estimating the effects of dual CYP3A4/CYP2C19 inhibitors (such as fluconazole [24]) under clinical scenarios, due to lack of further verification of fm_CYP2C19_ in vivo.

The model-predicted effects of CYP3A4 inducers (such as rifampin and efavirenz) on fedratinib PK also require further clinical verification, considering potential complex interplay between the inducers and fedratinib. In a recent retrospective survey on PBPK submissions to the US FDA [5], it was reported that PBPK-predicted effects of CYP3A4 inducers on CYP3A4 substrates agree well with clinical observations, but this finding may not be applicable to co-medications inducing enzymes other than CYP3A4 (e.g., rifampin as a dual CYP3A and CYP2C19 inducer [25]) or a substrate exhibiting time-dependent inhibitions on CYP3A4 (e.g., fedratinib as a mechanism-based inhibitor and inducer on CYP3A4 and CYP2C19).

The current PBPK model is not adequate to predict perpetrator effects of fedratinib on CYP2C19 and CYP2D6 substrates (such as omeprazole and metoprolol, respectively). The discrepancy between the model-predicted and clinically observed fedratinib effects on omeprazole and metoprolol may be a result of complex interaction profile of fedratinib in vivo on enzyme CYP2C19 and CYP2D6 that may not have been captured by the in vitro inhibitory profile used in the current PBPK model.

Additionally, the current modeling study focuses on the metabolic DDI risks of fedratinib other than transporter mediated DDI risks. Although the current PBPK model incorporates in vitro inhibition parameters of fedratinib on transporters including OCT1/2, BCRP, OATP1B1/3, and P-gp, the confidence in model-predicted DDI of fedratinib on the transporters remains low due to insufficient verification in vivo.

In future, the PBPK model of fedratinib will be further verified and refined using the additional clinical data obtained from the planned postmarketing studies. With the model improvement, the PBPK model is expected to more adequately evaluate DDI risks between fedratinib and dual CYP3A4/CYP2C19 inhibitors, between fedratinib and CYP3A4 inducers, and between fedratinib and transporter substrates.

## Conclusion

The PBPK model for fedratinib was developed to evaluate both victim and perpetrator DDI effects for the target metabolic pathways. The current model was found to capture clinical fedratinib PK profiles over the 300–500 mg dose range in both healthy subjects and MF patients, adequately assess DDI potential between fedratinib and CYP3A4 inhibitors, CYP3A4 substrates, CYP2C8 substrates, or CYP2C9 substrates. The PBPK simulations informed the drug label claims for fedratinib. Fedratinib dose should be reduced to 200 mg QD when strong CYP3A4 inhibitors (such as ketoconazole and itraconazole) are co-administered with fedratinib. In cases where a co-administered strong CYP3A4 inhibitor is discontinued, fedratinib dosage should be increased to 300 mg once daily during the first two weeks after discontinuation of the CYP3A4 inhibitor and then to 400 mg once daily thereafter as tolerated [1].

## Electronic supplementary material

Below is the link to the electronic supplementary material.

Supplementary file1 (DOCX 861 kb)
