# Supplementary material for: Physiologically based pharmacokinetic modeling to assess metabolic drug–drug interaction risks and inform the drug label for fedratinib
Source: Cancer Chemother Pharmacol. 2020 Sep 4;86(4):461–73. doi: 10.1007/s00280-020-04131-y (PMC7515950; doi:10.1007/s00280-020-04131-y)
Supplement: Supplementary file 1 — Supplementary file1 (DOCX 861 kb) [file 280_2020_4131_MOESM1_ESM.docx]

Supplemental Material of “Physiologically Based Pharmacokinetic Modeling to Assess Metabolic Drug-Drug Interaction Risks and Inform the Drug Label for Fedratinib”

Fan Wu,^1,2^, Gopal Krishna^2^, and Sekhar Surapaneni^1^

^1^ Nonclinical Research & Development, Bristol Myers Squibb, Summit, NJ, USA; ^2^ Clinical Pharmacology, Bristol Myers Squibb, Summit, NJ, USA

Keyword: Drug-drug interaction, PBPK, metabolic inhibition, Simcyp, fedratinib

# SM 1: Summary of preclinical and clinical studies used to support the model development

| **Study Number** | **Description** | **Reference** |
| --- | --- | --- |
| *Preclinical studies* | | |
| AIV0208 | This study was conducted to determine the apparent permeability coefficient of fedratinib (i.e., SAR302503A or TG101348), the effect of pH on the apparent permeability coefficient, and to evaluate the compound as a potential substrate and as a potential inhibitor of P-gp and MRP using the Caco-2/TC7 in vitro model of human intestinal barrier. The permeability coefficient was determined after addition of 20 μM SAR302503A (TG101348) in the apical chamber. The transport experiments were conducted in the presence of both protein (0.5 % BSA in the apical chamber and 5 % BSA in the basal chamber) and pH gradient (apical pH 6.5 and basal pH 7.4). The “apical to basal” transport of SAR302503A (TG101348) was measured by sampling aliquots from the basal chamber at t = 15, 30, 45 and 60 minutes. The recovery of SAR302503A (TG101348) was determined at the end of the incubation period. Samples were quantified by HPLC-MS/MS (see Section 10). The effect of pH was determined by measuring the Papp of SAR302503A (TG101348) with apical pH of 6.5 or 7.4. D-[1-^14^C]-mannitol (20 μM, 37 KBq/mL) and [4-^14^C]-testosterone (20 μM, 14.8 KBq/mL) were used as low and high permeability reference compounds, respectively. | Internal study report |
| IHH0014 | This study was conducted to evaluate the potential induction effect of fedratinib on CYP1A, CYP2B6 and CYP3A enzyme activities and gene expression in primary cultures of human hepatocytes. Cryopreserved human hepatocytes were obtained from Kaly-Cell (Strasbourg, France), Invitrogen and BD Biosciences. After assessment of cellular viability using the trypan blue exclusion test, human hepatocytes were seeded onto collagen-coated plates. For the measurement of CYP enzyme activities and gene expression, hepatocytes were seeded onto collagen-coated 24-well plates at 400x10^3^ viable cells/well. The incubation medium consisted of HMM medium supplemented with ITS (1%), gentamycin (50 μg/mL) and dexamethasone (0.1 μM). Hepatocytes were cultured in a 37°C thermostatically controlled incubator with 5% CO_2_ and 95% hygrometry. | Internal study report |
| LPR1082 | This study was conducted to determine in vitro the binding of [^14^C]-fedratinib to human plasma proteins and to specific human plasma proteins (α_1_-acid glycoprotein and serum albumin). [^14^C]-SAR302503 was dissolved in an adequate amount of ethanol to obtain a stock solution with a concentration of 1 mg/mL. The radiochemical purity of each stock solution was checked using HPLC method used by ICMS unit (HPLC with on-line radioactivity detection) prior to experiments. The non-specific adsorption was studied using dialysis conditions (plasma/buffer) at 1000 ng/mL. After dialysis (incubation time of 4 h), apparatus was rinsed with buffer, then with a solution of ethanol. For protein binding, the pH of human plasma, AAG and HSA solutions were measured using calibrated pH meter before the addition of [^14^C]-SAR302503. Samples with a pH value outside the range 7 to 8 were not used. Samples of human plasma, AAG and HSA solutions were transferred to the Medical Analysis laboratory Dullin (7 rue Salvador Allende, Bagneux, France) for protein concentrations measurement to ensure that there were within normal limits (between 50-80 mg/mL for human plasma proteins and close to 0.8 g/L and 40 g/L, for AAG and HSA respectively.). The determination of binding to human plasma proteins and to human specific proteins (HSA and AAG) was performed at 50, 500, 1000, 5000 and 10 000 ng/mL. | Internal study report |
| MEH0082 | This study was conducted to determine the metabolic profiles and to identify the chemical structures of the main metabolites of fedratinib after a single oral administration of [^14^C]-fedratinib to healthy male subjects using samples (plasma, urine and feces) collected from the study BEX12257. Aliquots of pooled feces homogenates were digested with soluene-350 during 2 hours at 37°C. Aliquots of sample pools of plasma, urine and their corresponding extracts in addition to the digested feces homogenates were assayed for radioactivity content by liquid scintillation counting (LSC) using Perkin Elmer Tricarb 2900 and 3100 after addition of an appropriate scintillation  fluid. | Internal study report |
| MIH0887 | This study was conducted to investigate potential inhibition of CYP enzymes and to determine corresponding inhibition constants by fedratinib in human liver microsome reaction mixtures.   - To determine apparent Ki values of reversible inhibition, HLM reaction mixtures were prepared using a minimum of four CYP-selective substrate concentrations and multiple SAR302503 concentrations. Reaction mixtures were also prepared at each substrate concentration, but without added SAR302503. Substrate concentrations were chosen to bracket the apparent Km concentration, with one concentration the same as that used for IC50 determination. SAR302503 concentrations were chosen to observe approximately 10% to 90% inhibition estimated from the IC50 value determined for each inhibited CYP isoform. Reaction mixture components were incubated concurrently under conditions specific for each CYP activity assay. All reaction mixtures were prepared in duplicate. - To determine enzyme kinetic parameters, K_inact_ and K_I_ of irreversible inhibition, reaction mixtures were prepared in Kphos with HLM, an NADPH generating system and SAR302503 at multiple concentrations. The reaction mixtures were pre-incubated at 37°C for specific time periods. At the end of the pre-incubation period, 20 μL of each reaction mixture were transferred into a second set of reaction mixtures containing CYP selective substrate and fresh NADPH generating system in Kphos. In order to reduce the effect of residual reversible inhibition, probe substrate concentrations were 4-fold to 10-fold higher than their respective apparent Km concentrations. The reaction mixtures were incubated for an additional period time specific to each CYP activity assay. | Internal study report |
| MIH0888 | This study was conducted to investigate the in vitro intrinsic clearance of clearance using fresh or cryopreserved human hepatocytes in primary culture, to determine the contribution of metabolic enzymes, and to monitor potential metabolite formulation using human hepatocytes. Stock solutions of SAR302503 were prepared at 0.1 mM, 1 mM and 10 mM in DMSO and stored at +4°C. Working solutions were prepared by 1000-fold dilution of stock solutions in incubation medium. 100 μL of these working solutions were put on cells to achieve the final concentrations of 0.1 μM, 1 μM and 10 μM (52, 525 and 5247 ng/mL of SAR302503, respectively). Stock solutions were prepared in DMSO at 3 mM for ketoconazole, quinidine and omeprazole or 1 M for aminobenzotriazole (ABT) and methimazole, and stored at -20°C. Just prior to hepatocyte incubation, 10 μL of these stock solutions (or DMSO for control) were added in 10mL of appropriate working solutions of test compound, to achieve the final concentrations of 3 μM for ketoconazole, quinidine and omeprazole or 1 mM for ABT and methimazole. The final solvent concentration in the incubation medium was 0.2% in all cases. Four cryopreserved human hepatocyte preparations were plated in the lab. They were supplied by Celsis-InVitroTechnologies (Brussels, Belgium) and Invitrogen-GIBCO (Cergy Pontoise, France). Seeding was performed following recommendations of each supplier. Cells were rapidly thawed at 37°C and washed to remove DMSO. After centrifugation, pellets were resuspended in incubation medium. Viability was assessed by trypan blue exclusion test and hepatocyte concentration was adjusted to dispense 168000 cells per well. Hepatocytes were seeded onto collagen-coated 48-well plates. They were incubated 4 hours before changing culture medium. Cells were let to recover one night in the incubator. This study was conducted only on cryopreserved hepatocytes. Hepatocyte metabolic capacities were determined for each preparation on CYP3A, CYP2D6, CYP2C9 and CYP1A activities. Results were compared to mean historical values. Quantification of midazolam and marker metabolites was performed using LC-MS/MS. | Internal study report |
| PDM-07-10348-059 | This study was conducted to evaluate the biliary excretion of fedratinib in rats following a single intravenous (iv) dose of 5 mg/kg. Male Sprague Dawley rats (N=5) with surgically implanted bile duct cannulas and jugular vein cannulas were obtained from Harlan (Indianapolis, IN). Rats were individually housed in metabolism cages and jugular vein cannulas were flushed with heparinized saline. In a non-fasted state, rats were administered iv via a single injection in the tail vein with TG101348 formulation at 5 mg/kg. Free flowing bile was collected in the intervals of 0 to 2 hours, 2 to 4 hours, 4 to 7 hours, and 7 to 24 hours. Bile samples were collected from only 3 rats. | Internal study report |
| QUA-PG-2011-20593 | This study was conducted to support drug product development, to evaluate the impact of drug substance physical quality on dissolution and on drug product behavior in the conditions of clinical administration. | Internal study report |
| TRE0075 | This study was conducted to investigate SAR302503 as a substrate and an inhibitor of the human hepatic uptake transporters hOATP1B1 and hOATP1B3 using HEK^TR^, HEK^TR^-hOATP1B1 and HEK^TR^-hOATP1B3 cell lines in vitro. All inhibition experiments were performed with HBSS assay buffer (Gibco BRL, containing 10 mM HEPES at pH 7.4) in 96-well plates in quadruplet at 37°C. The buffer volume was 50 μL in each well. Overexpressing cells and control cells were always treated in parallel. Cells were washed with 200 μL HBSS assay buffer (37°C) and then incubated in the presence of the radiolabeled substrate including various inhibitor concentrations (50 μL/well; assay designs are described below for each cell line) at 37°C. Uptake was stopped by adding 150 μL/well icecold HBSS assay buffer. Solution was removed and cells were washed two times with 200 μL/well ice-cold HBSS assay buffer. After complete removal of the washing solution 100 μL/well scintillation liquid (Microscint^TM^ 40) were added and cells were lysed for 30 min under a continuous orbital agitation (500 rpm). Radioactivity in each well was determined by liquid scintillation counting (TopCount NXT, Perkin Elmer Life Sciences).   - hOATP1B1: probe substrate – 1 μM [^3^H]E17βG (10 min) probe inhibitor – 2 μM and 10 μM Rifampicin - hOATP1B3: probe substrate – 0.5 μM [^3^H]CCK8 (5 min) probe inhibitor – 2 μM and 10 μM Rifampicin - Concentration of the test compound: SAR302503: 0, 1, 5, 10 and 50 μM for an initial screening experiment 0, 0.1, 0.3, 0.7, 1, 3, 7, 10, 30 and 100 μM for determination of IC_50_ values | Internal study report |
| TRI0013 | This study was conducted to investigate fedratinib as an inhibitor of the renal uptake transporters hOAT3, hOCT1 and hOCT2 and the efflux transporter hBCRP using the CHO^Fi^, CHO^Fi^-hOAT3, CHO^Fi^-hOCT1 and CHO^Fi^-hOCT2 cell lines in vitro. All inhibition experiments were performed with HBSS assay buffer (Gibco BRL, containing 10 mM HEPES at pH 7.4) in 96-well plates in quadruplet, at 37°C. The buffer volume was 50 μL in each well. Overexpressing cells and control cells were always treated in parallel. In all experiments with CHOFi-hOAT3 cells, a stimulation of hOAT3 activity was initiated with 100 μL/well 500 μM Glutaric acid for at least 60 min. Cells were washed with 200 μL HBSS assay buffer (37°C) and then incubated in the presence of the radiolabeled substrate including various inhibitor concentrations (50 μL/well; assay designs are described below for each cell line) at 37°C. Uptake was stopped by adding 150 μL/well ice-cold HBSS assay buffer (containing 300 μM quinine or 500 μM probenecid for all experiments analyzing hOCT2 and hOAT3, respectively). Solutions were removed and cells were washed two times with 200 μL/well ice-cold HBSS assay buffer. After complete removal of the washing solutions 100 μL/well scintillation liquid (Microscint^TM^ 40) were added and cells were lysed for 30 min under continuous orbital agitation (500 rpm). Radioactivity in each well was determined by liquid scintillation counting (TopCount NXT, Perkin Elmer Life Sciences).   - hOAT3: probe substrate – 0.05 μM [^3^H]Estrone-3-sulfate (0.5 min) probe inhibitor – 10 μM and 50 μM Probenecid - hOCT1: probe substrate – 25 μM [^14^C]TEA (15 min) probe inhibitor – 100 μM and 500 μM Quinine - hOCT2: probe substrate – 25 μM [^14^C]Metformin (1 min) probe inhibitor – 10 μM and 50 μM Quinine - Concentration of the test compound: SAR302503: 0, 1, 5, 10 and 50 μM (for a first screening experiment) 0, 0.1, 0.3, 0.7, 1, 3, 7, 10, 30 and 100 μM (full IC50 determination) | Internal study report |
| *Clinical studies* | | |
| ALI13451 | This phase I study was conducted to investigate the effect of food intake on the pharmacokinetics (PKs) and tolerability of single dose fedratinib (500 mg under fasted or fed [low- or high-fat breakfast] conditions) in healthy male subjects. See Reference [1] for the detailed information of the ethics committee. Written informed consent was obtained from all subjects. | [1] |
| ARD11936 | A Phase 2 study was conducted to investigate the efficacy and safety of orally administered fedratinib (300, 400, and 500 mg QD) in patients with intermediate-2 or high-risk primary myelofibrosis, post-polycythemia vera myelofibrosis, post-essential thrombocythemia myelofibrosis with splenomegaly. The protocol was approved by the institutional review boards (IRBs) at all participating sites (University of California, San Deigo IRB, University of Michigan IRB, Mayo Clinic IRB, and Shulman Associates Institutional Review Board, Cincinnati, OH) for review and written approval. Written informed consent was obtained from all subjects. | [2] |
| BDR12462 | This clinical study was conducted to investigate bioequivalence of fedratinib in the formulation of 500 mg tablets versus 500 mg capsules. The protocol was approved by the institutional review boards (IRBs) at all participating sites (Independent Review Board, Inc., Sunrise, FL) for review and written approval. Written informed consent was obtained from all subjects. | [3] |
| BEX12257 | This clinical study was conducted to investigate absorption, distribution, metabolism, and distribution (ADME) of fedratinib following a single 200 mg dose in healthy subjects.  The protocol was approved by the institutional review boards (IRBs) at all participating sites (Independent Review Board, Inc., Plantation, FL) for review and written approval. Written informed consent was obtained from all subjects. | [3] |
| FED12258 | This phase I study was conducted to investigate the effect of food intake on the pharmacokinetics (PKs) and tolerability of single dose fedratinib (100 mg or 500 mg under fasted or fed [high-fat breakfast] conditions) in healthy male subjects. Written informed consent was obtained from all subjects. | [1] |
| INT12497 | This clinical study was conducted to investigate drug-drug interaction between fedratinib (500 mg QD, 15 days) and CYP probe cocktail (2C19, 2D6 and 3A4) in patients with solid tumor. See Reference [4] for the detailed information of the ethics committee. Written informed consent was obtained from all subjects. | [4] |
| INT12893 | This clinical study was conducted to investigate drug-drug interaction between fedratinib (300 mg, single dose) and ketoconazole (CYP3A4 strong inhibitor, 200 mg BID) in healthy subjects. See Reference [5] for the detailed information of the ethics committee. Written informed consent was obtained from all subjects. | [5] |
| TDU12620 | This clinical study was conducted to investigate tolerability and pharmacokinetics of ascending single doses of fedratinib in healthy male subjects. See Reference [6] for the detailed information of the ethics committee. Written informed consent was obtained from all subjects. | [6] |

# SM 2: Descriptions of drug properties and model input parameters

## Material

The molecular weight of fedratinib is 615.62 g/mol (or 524.68 g/mol in the free form) [7] and its chemical structure of fedratinib is shown in Figure 1.


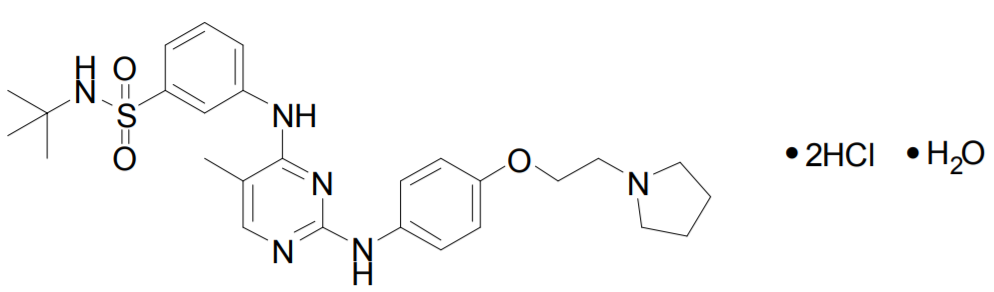


Figure 1: The Chemical Structure of Fedratinib

## Input Parameters for the PBPK Model

The values of input parameters for the PBPK model were summarized in Table 1. The preclinical and clinical studies used to derive the parameter values were briefly described in Supplemental Material (SM1).

### Physicochemical and Blood Binding Parameters

The physicochemical properties and blood binding parameters of fedratinib including molecular weight, logP, pKa, fu, and B/P were experimentally measured and listed in Table 1.

Table 1: Input Values of Physicochemical and ADME Parameters for Fedratinib

| Parameter | Value | Reference |
| --- | --- | --- |
| *Physicochemical properties and blood binding* | | |
| MW | 525 | Study QUA-PG-2011-20593 |
| LogP_o:w_ | 3.5 | Study QUA-PG-2011-20593 |
| pK_a_ | 6.4 (base) 9.6 (base) | Study QUA-PG-2011-20593; free form of fedratinib is a diprotic base, with 6.4 and 9.6 registered for pKa1 and pKa2 in Simcyp®, respectively |
| fu | 0.05 | Study LPR1082; mainly bound to α_1_-acid glycoprotein |
| B/P | 0.7 | Study BEX12257 |
| *Absorption* | | |
| fa | 0.65 | Analyses of data from Study BEX12257 and MEH0082 |
| ka (h^-1^) | 0.22 | Compartmental PK modeling of data from  TDU12620, BDR12462, FED12258, ALI13451 |
| Caco-2 P_app_ (10^-6^ cm/s) | 0.21 to 17.8 | Study AIV0208; 0.21 measured at Apical pH: Basolateral pH = 6.5:7.4; 17.8 measured at Apical pH: Basolateral pH = 7.4:7.4; used to predict the range of Q_Gut_ values |
| fu_Gut_ | 1 | Default |
| Q_Gut_ (L/h) [range] | 6.5 [1.34 to 11.5] | User input value as the mean of the minimum and maximum Q_Gut_ values derived from Caco-2 P_app_ values within Simcyp® |
| *Distribution* | | |
| Vss (L/kg) | 4.1 | Analyses of data from TDU12620, BDR12462, FED12258, ALI13451 |
| Vsac (L/kg) | 3.5 |  |
| Q (L/h) | 11 |  |
| *Metabolism* | | |
| CL_int,CYP3A4_ (mL/min/pmol of isoform) | 0.610 | Retrograde modeling of clinical and preclinical data with further refinement using the clinical repeated dose PK data (see Supplemental Material SM8) |
| CL_int,CYP2D6_ (mL/min/pmol of isoform) | 0.258 |  |
| CL_int,CYP2C19_ (mL/min/pmol of isoform) | 1.25 |  |
| Additional HLM clearance (mL/min/mg protein) | 0 |  |
| *Excretion* | | |
| CL_R_ (L/h) | 1.67 | User input estimated from BEX12257, MEH0082 |
| CL_Add_ (L/h) | 0.67 | User input estimated from PDM-07-101348-059 |

B/P = blood/plasma concentration ratio; CL_Add_ = additional systemic clearance; CL_int_ = intrinsic metabolic clearance; CL_R_ = renal clearance; fa = fraction of absorbed dose; fu = unbound fraction in plasma; fu_Gut_ = unbound fraction in enterocytes; ka = first-order absorption constant; MW = molecular weight; Papp = apparent permeability coefficient; pK_a_ = acid dissociation constant; Q = inter-compartment clearance; Q_Gut_ = a nominal flow in gut model; Vsac = volume of the single adjustment compartment; Vss = steady-state volume of distribution.

### Absorption Parameters

The first-order absorption ka value was estimated to be ~0.22 h^-1^ based on compartmental modeling of mean PK data obtained from healthy subjects given single doses of 500 mg fedratinib (see Table 1 in Supplemental Material SM 2). The fa value was estimated based on data obtained from the mass balance and metabolic profiling studies conducted in healthy subjects. By combining the estimated excretion percentages of the parent compound in urine, feces, and bile, the unabsorbed dose was estimated to be ~35% and thus fraction of absorption (fa) was estimated to be ~0.65 (see Figure 2 and “Excretion Parameters” subsection).

Intestinal permeability of fedratinib was suggested to be concentration- and pH-dependent based on the *in vitro* Caco-2 permeability assay, from 0.21×10^-6^ cm/s (measured at 40 μM fedratinib and Apical pH: Basolateral pH = 6.5:7.4) to 17.8×10^-6^ cm/s (measured at 100 μM fedratinib and Apical pH: Basolateral pH = 7.4:7.4). Accordingly, the human intestinal P_eff_ was predicted in Simcyp^®^ to range from 0.16×10^-6^ cm/s to 2.6×10^-6^ cm/s, and the hybrid intestinal blood flow parameter (Q_Gut_) from 1.34 L/h to 11.5 L/h. By default, the Q_Gut_ is set to be the mean value (i.e., 6.5 L/h) in the PBPK model of fedratinib.

In clinically relevant dose range of 300 to 500 mg, fedratinib exposure was roughly dose proportional in MF patients, indicating no non-linearity due to any efflux [2, 3]. As evidenced from human mass balance study, absorbed fedratinib was extensively metabolized and excreted and the excretion of intact drug is not a significant pathway of excretion [3]. Overall, P-gp mediated drug efflux is not expected to impact bioavailability and PK properties of fedratinib as a fast absorbing and large-dose drug [8]. Therefore, intestinal P-gp kinetics was not incorporated into the model and the Q_Gut_ is set to be the mean value (i.e., 6.5 L/h) in the PBPK model of fedratinib.

### Distribution Parameters

The minimal PBPK model within Simcyp^®^ was used to describe the distribution of fedratinib. The Vss, Vsac, and Q values were estimated using the compartmental PK modeling approach (see Table 3 in Supplemental Material SM 3) and further refined within Simcyp^®^ based on the model-predicted bioavailability (F) using the following equations:

$\boldsymbol{Vss}=\left( \boldsymbol{V}/\boldsymbol{F}+\boldsymbol{V}\boldsymbol{2}/\boldsymbol{F} \right)\cdot\boldsymbol{F}$ (1)

$\boldsymbol{Vsac}=\boldsymbol{V}\boldsymbol{2}/\boldsymbol{F}\cdot\boldsymbol{F}$ (2)

$\boldsymbol{Q}=\boldsymbol{CL}\boldsymbol{2}/\boldsymbol{F}\cdot\boldsymbol{F}$ (3)

where V/F refers to apparent volume of distribution of the central compartment, V2/F refers to apparent volume of distribution of the peripheral compartment, CL2/F refers to apparent inter­compartment clearance, and F refers to bioavailability predicted to be ~0.36 in a representative healthy subject with the body weight of ~81 kg given a single 500 mg dose of fedratinib.

Using the above equations, as the V/F, V2/F, and CL2/F were estimated to 107 L, 797 L, and 30.8 L/h by the compartmental PK modeling of the clinical oral single-dose PK data in healthy subjects (see Supplemental Material SM3), Vss, Vsac, and Q were calculated to be ~4.1 L/kg, ~3.5 L/kg, and ~11 L/h, respectively, assuming the oral bioavailability (F) of ~0.36 and body weight of ~81 kg. The estimated values of Vss, Vsac, and Q were directly supplied to the Distribution module within Simcyp® without further modifications. Note that since the intravenous (IV) PK data are not available in humans for fedratinib, the distribution parameters include Vss estimated from the oral human PK data may not be accurate and will be subject to further verification if the human IV PK data of fedratinib or other relevant clinical information are available in the future.

### Metabolism Parameters

The retrograde modeling tool provided within Simcyp^®^ was applied to estimate the CL_int_ for CYP enzymes based on the CL_po_ estimated via compartmental PK modeling (Supplemental Material SM3) and the hepatic metabolic clearance percentages determined from cryopreserved human hepatocytes incubated with 1 μM fedratinib, which were 17%, 2%, and 63% for CYP2C19, CYP2D6, and CYP3A, respectively. As CYP3A4 is the dominant form of CYP3A in the liver, all metabolic contribution from CYP3A was assigned to CYP3A4 in this PBPK model. The default “Healthy Volunteers” population was used for the retrograde modeling. The hepatic metabolic CL percentages required for retrograde modeling are estimated from in vitro experimental data obtained from cryopreserved human hepatocytes incubated with 1 μM fedratinib. The F_g_ value required for retrograde modeling was initially set as default 1 and then adjusted to ~0.8 based on model prediction. The CL_po_ value used for retrograde calculation of fedratinib was initially set as the CL/F value estimated using the compartmental PK modeling of the mean PK profiles in healthy subjects given single doses of 500 mg fedratinib and then updated to 48 L/h so that the derived CL_int_ values result in agreement between the predicted dose-normalized AUC and the observation (i.e., 30 ng·h/(mL·mg)) reported for healthy subjects given single doses above 100 mg fedratinib [3]. Following an iterative process and manual parameter sensitivity analyses (see Supplemental Material SM8), the intrinsic clearance values were determined with CL_int,CYP3A4_ = 0.610 μL/min/pmol of isoform, CL_int,CYP2D6_ = 0.258 μL/min/pmol of isoform, CL_int,CYP2C19_ = 1.25 μL/min/pmol of isoform, and additional HLM clearance = 0 μL/min/mg protein, respectively, for healthy subjects. The clearance values derived above for fedratinib in healthy subjects were assumed to be the same for cancer patients including myelofibrosis (MF) patients and patients with refractory solid tumors.

### Excretion Parameters

The percent contributions of the parent compound (i.e., fedratinib) to administered dose (2.9% in urine and 23.3% in feces) were obtained from the human ^14^C-mass balance and profiling study and then normalized by the total percent activity of administered dose over 0-192/240h, i.e., 62.7%, thus accounting for the remaining radioactivity after 192/240 hr excreted from the human body. Following the normalization, excretion percentages of fedratinib were estimated to be ~5% and ~37% of the administered dose in urine and feces, respectively. Assuming the dose-normalized AUC of 30 ng·h/(mL·mg) [3] or the corresponding CL/F of 33.3 L/h, the CL_R_ was estimated to be ~1.67 L/h.

The biliary excretion of unchanged drug accounts for 2.3% of the total intravenous dose in bile-duct cannulated (BDC) rats. Thus, given the physicochemical properties and metabolic profile, the biliary excretion percentage was assumed to be ~2% in humans following an oral dose of fedratinib, leading to an estimation of ~0.67 L/h for the additional systemic clearance (CL_Add_) using CL/F of 33.3 L/h derived from the clinical observed dose-normalized AUC [3].


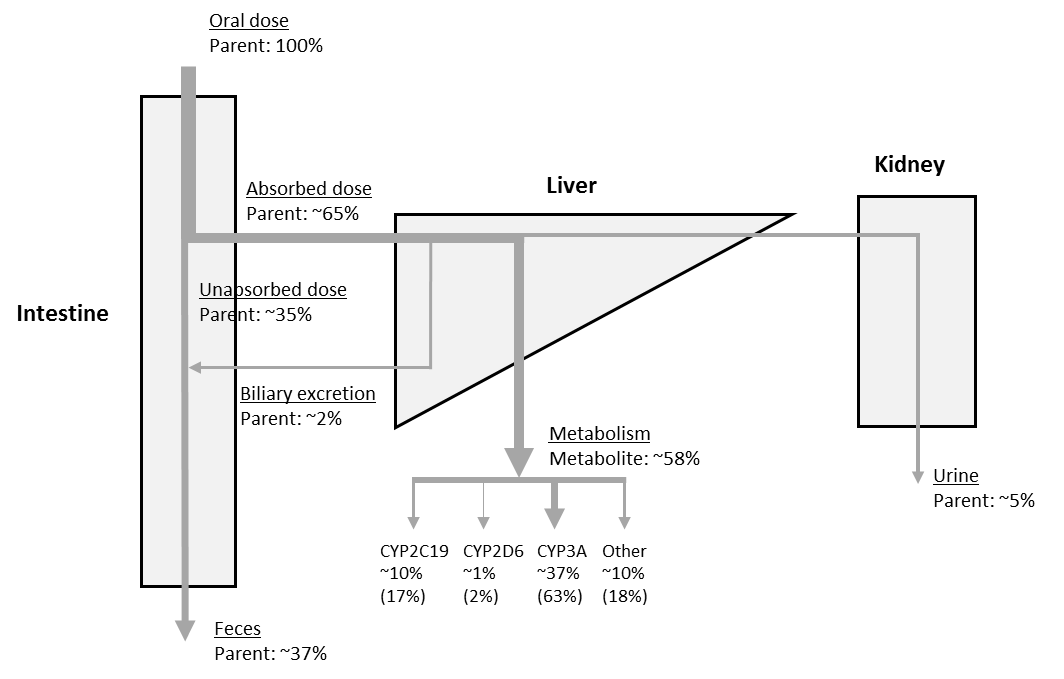


Figure 2. Proposed Elimination Pathways of Fedratinib in Humans. The percentages of the total oral dose as the parent compound or metabolites were labeled along with the elimination or metabolism routes. The relative contributions to the total metabolic clearance were included in the parentheses for each metabolic enzyme. The relative contribution of the other hepatic metabolic pathways was incorporated into the CYP3A pathway in the final PBPK model. The thickness of lines was adjusted to approximately reflect the relative contributions of the corresponding pathways.

### Interaction Parameters

The CYP enzyme reverse inhibition and mechanism-based inhibition (MBI) parameters were estimated in the human liver microsome (HLM) incubation *in vitro* studies. The reported reversible inhibition parameter (i.e., apparent Ki) values for CYP2C8, CYP2C9, and CYP2D6, along with the MBI parameters (i.e., K_I_ or K_app_ and k_inact_) for CYP2C19 and CYP3A4, were supplied to the PBPK DDI model (see Table 2). The apparent Ki value for CYP3A4 was approximated as IC_50_/2, with the IC_50_ value (i.e., 19.1 μM) estimated from the CYP3A assay using midazolam as the substrate in absence of NADPH during pre-incubation.

The induction effect of fedratinib on CYP3A gene expression was identified using human hepatocytes in primary culture. The reported kinetic data, i.e., concentration-dependent fold induction of CYP3A mRNA levels, were fitted to estimate the Ind_max_ and IndC_50_ values for fedratinib with calibration against the induction effect observed for the positive control compound rifampin [9].

Table 2: Input Values of Drug Interaction Parameters for Fedratinib

| Parameter | Value | Reference |
| --- | --- | --- |
| *Enzyme inhibition* | | |
| CYP2C8 Ki (μM) | 50.4 | MIH0887; HLM incubation study in vitro |
| CYP2C9 Ki (μM) | 26.1 | MIH0887; HLM incubation study in vitro |
| CYP2D6 Ki (μM) | 10.1 | MIH0887; HLM incubation study in vitro |
| CYP2C19 K_I_ or K_app_ (μM) | 112 | MIH0887; HLM incubation study in vitro |
| CYP2C19 k_inact_ (h^-1^) | 2.01 | MIH0887; HLM incubation study in vitro |
| CYP3A4 Ki (μM) | 9.55 | MIH0887; HLM incubation study in vitro (assuming Ki = IC_50_/2) |
| CYP3A4 K_I_ or K_app_ (μM) | 1.57 | MIH0887; HLM incubation study in vitro |
| CYP3A4 k_inact_ (h^-1^) | 2.58 | MIH0887; HLM incubation study in vitro |
| *Enzyme induction* | | |
| CYP3A4 IndC_50_ (μM) | 5.3 | IHH0014; Human hepatocyte culture study in vitro |
| CYP3A5 Ind_max_ | 78.0 | IHH0014; Human hepatocyte culture study in vitro |
| *Transporter inhibition* | | |
| OCT1 Ki (μM) | 5.59 | TRI0013; Human OCT1 cell culture study in vitro |
| OCT2 Ki (μM) | 0.71 | TRI0013; Human OCT2 cell culture study in vitro |
| BCRP Ki (μM) | 27.8 | TRI0013; Human BCRP vesicle-based study in vitro |
| OATP1B1 Ki (μM) | 14.6 | TRE0075; Human OATP1B1 cell culture study in vitro |
| OATP1B3 Ki (μM) | 8.4 | TRE0075; Human OATP1B3 cell culture study in vitro |
| P-gp Ki (μM) | 10.0 | AIV0208; In vitro permeability and P-gp interaction study |

HLM = human liver microsomes; IC_50_ = compound concentration that supports half maximal inhibition; IndC_50_ = compound concentration that supports half maximal induction; Ind_max_ = maximal fold induction over vehicle; Ki = concentration of inhibitor that supports half maximal inhibition; K_I_ or K_app_ = concentration of mechanism-based inhibitor associated with half maximal inactivation rate; k_inact_ = inactivation rate of the enzyme.

As observed in studies *in vitr*o, fedratinib was found to inhibit various transporters, including OCT1, OCT2, BCRP, OATP1B1, and OATP1B3, and P-gp. The reported IC50 values were converted to Ki values using the Cheng-Prusoff equation [10]:

$\boldsymbol{Ki}={\boldsymbol{IC}_{\boldsymbol{50}}}/\left( \boldsymbol{1}+\left[ \boldsymbol{S} \right]/\boldsymbol{K}_{\boldsymbol{m}} \right)$ , (4)

where [S] denotes the probe substrate concentrations in the experiments and K_m_ denotes the substrate concentration at which the maximal reaction velocity is achieved, which were obtained from literature. Additional details of deriving transporter Ki for fedratinib is included in Supplemental Material SM11.

# SM 3: Compartmental PK modeling to derive initial minimal PBPK parameters

The temporal profiles of mean plasma concentration of fedratinib were collected from the clinical studies ([TDU12620, BDR12462, FED12258](#references), and [ALI13451](#references)) conducted in healthy volunteers (HVs) administered with single doses of 500 mg fedratinib. The compartmental PK modeling was conducted in Phoenix^®^/NLME (V7, Certara, Princeton, NJ). A two-compartment PK structural model with the additive error model was identified to successfully describe the PK data. The “naïve pooled” algorithm provided in Phoenix was used for parameter estimation. The obtained compartment PK parameters were summarized in Table 3.

Table 3: Compartmental PK Model Parameters of Fedratinib

| Parameter | Estimate |
| --- | --- |
| ka (h^-1^) | 0.221 |
| V/F (L) | 107 |
| V2/F (L) | 797 |
| CL/F (L·h^-1^) | 27.2 |
| CL2/F (L·h^-1^) | 30.8 |

CL/F = bioavailability-normalized central plasma clearance (i.e., oral clearance, CL_po_); CL2/F = bioavailability-normalized inter-compartment clearance (i.e., Q/F); ka = first-order absorption constant; V/F = bioavailability-normalized central volume of distribution; V2/F = bioavailability-normalized peripheral volume of distribution.

# SM 4: Simcyp^®^ model inputs of the fedratinib PBPK model

The Simcyp® model inputs of the fedratinib PBPK model was included below.

| Compound Name | fedratinib_middle_out_retrograde_v2 |
| --- | --- |
| Version number | Not applicable |
| Molecule Type | Small Molecule |
| Route | Oral |
| Dose Units | Dose (mg) |
| Dose | 500.000 |
| Start Day | 1.000 |
| Start Time | 9h0m |
| Dosing Regimen | Single Dose |
|  |  |
| PhysChem and Blood Binding |  |
|  |  |
| Mol Weight (g/mol) | 525.000 |
| log P | 3.500 |
| Compound Type | Diprotic Base |
| pKa 1 | 6.400 |
| pKa 2 | 9.600 |
| BP input type | User |
| B/P | 0.700 |
| Haematocrit | 45.000 |
| fu Input | User |
| fu | 0.050 |
| Reference Binding Component | AGP |
| Protein Reference Conc (g/L) | 0.811 |
| % Bound to Lipoprotein | 0.000 |
| % Bound to Lipoprotein (CV %) | 0.000 |
|  |  |
| Absorption |  |
|  |  |
| Absorption Model | 1st order |
| Input type | User |
| fa | 0.650 |
| CV fa (%) | 30.000 |
| ka (1/h) | 0.220 |
| CV ka (%) | 30.000 |
| lag time (h) | 0.000 |
| CV lag time (%) | 30.000 |
| fu(Gut) input type | User |
| fu(Gut) | 1.000 |
| Q(Gut) Input | User |
| Q(Gut) (L/h) | 6.500 |
| CV Q(Gut) (%) | 30.000 |
| Peff,man Type | n/a |
| Permeability Assay | PCaco-2 |
| Apical pH : Basolateral pH | 7.4 : 7.4 |
| Activity | Passive & Active |
| PCaco-2(10E-06 cm/s) | 17.800 |
| Reference Compound | Propranolol |
| Reference Compound Value (10E-06 cm/s) | 43.000 |
| Scalar | 1.000 |
|  |  |
| Distribution |  |
|  |  |
| Distribution Model | Minimal PBPK Model |
| SAC kin (1/h) | 0.000 |
| SAC kout (1/h) | 0.000 |
| SAC CLin (L/h) | 1.00E-05 |
| SAC CLout (L/h) | 1.00E-05 |
| SAC Q (L/h) | 11.000 |
| Volume [Vsac] (L/kg) | 3.50E+00 |
| Vss input type | User |
| Vss (L/kg) | 4.100 |
| CV Vss (%) | 30.000 |
| Liver input type | User |
| Liver Kp | 1.000 |
|  |  |
| Elimination |  |
|  |  |
| Clearance Type | Enzyme Kinetics |
| FI Correction | Not used |
| PLR Correction | Not Used |
| In vitro metabolic system | Recombinant |
|  |  |
| Pathway | Pathway 1 |
| Enzyme | CYP3A4 |
| CLint (µL/min/pmol) | 0.610 |
| fu mic | 1.000 |
|  |  |
| Pathway | Pathway 1 |
| Enzyme | CYP2C19 |
| CLint (µL/min/pmol) | 1.253 |
| fu mic | 1.000 |
|  |  |
| Pathway | Pathway 1 |
| Enzyme | CYP2D6 |
| CLint (µL/min/pmol) | 0.258 |
| fu mic | 1.000 |
|  |  |
| Use Allelic variants for Enzyme 1 | No |
| Enzyme | CYP2C9 |
|  |  |
| Use Allelic variants for Enzyme 2 | No |
| Enzyme | CYP1A2 |
|  |  |
| Ontogeny Profile | No Profile Used |
| Biliary CLint (Hep) (µL/min/10^6^) | 0.000 |
| CV Biliary CLint (Hep) (%) | 30.000 |
| Ontogeny Profile | No Profile Used |
| Active Uptake into Hepatocyte | 1.000 |
| CL R (L/h) | 1.670 |
| Additional Systemic Clearance (L/h) | 0.670 |
| Additional Systemic Clearance CV (%) | 30.000 |
| Ontogeny Profile | No Profile Used |
|  |  |
| CYPs and/or UGTs Interaction |  |
|  |  |
| Enzyme | CYP2C8 |
| Ki (µM) | 50.400 |
| fu mic | 1.000 |
|  |  |
| Enzyme | CYP2C9 |
| Ki (µM) | 26.100 |
| fu mic | 1.000 |
|  |  |
| Enzyme | CYP2C19 |
| MBI Kapp (µM) | 112.000 |
| MBI Kinact (1/h) | 2.010 |
| MBI fu mic | 1.000 |
|  |  |
| Enzyme | CYP2D6 |
| Ki (µM) | 10.100 |
| fu mic | 1.000 |
|  |  |
| Enzyme | CYP3A4 |
| Ki (µM) | 9.550 |
| fu mic | 1.000 |
| MBI Kapp (µM) | 1.570 |
| MBI Kinact (1/h) | 2.580 |
| MBI fu mic | 1.000 |
| Ind max | 78.000 |
| CV (%) | 30.000 |
| MIA (pmol/mg microsomal protein) | 19587.438 |
| Ind C50 (µM) | 5.300 |
| CV (%) | 30.000 |
| fu inc | 1.000 |
| γ | 1.000 |
|  |  |
| Co-administration of Multiple Inducers/Suppressors | Default |
| Co-administration of Multiple UGT Inducers/Suppressors | Default |
|  |  |
| Transporters Interaction |  |
|  |  |
| Organ/Tissue | Gut |
| Transporter | SLC22A1 (OCT1) |
| Ki (µM) | 5.590 |
| fuinc (Ki) | 1.000 |
| Transporter | ABCB1 (P-gp/MDR1) |
| Ki (µM) | 10.000 |
| fuinc (Ki) | 1.000 |
| Transporter | ABCG2 (BCRP) |
| Ki (µM) | 27.800 |
| fuinc (Ki) | 1.000 |
|  |  |
| Organ/Tissue | Liver |
| Transporter | SLCO1B1 (OATP1B1) |
| Ki (µM) | 14.600 |
| fuinc (Ki) | 1.000 |
| Transporter | SLCO1B3 (OATP1B3) |
| Ki (µM) | 8.400 |
| fuinc (Ki) | 1.000 |
| Transporter | SLC22A1 (OCT1) |
| Ki (µM) | 5.590 |
| fuinc (Ki) | 1.000 |
| Transporter | ABCB1 (P-gp/MDR1) |
| Ki (µM) | 10.000 |
| fuinc (Ki) | 1.000 |
| Transporter | ABCG2 (BCRP) |
| Ki (µM) | 27.800 |
| fuinc (Ki) | 1.000 |
|  |  |
| Organ/Tissue | Kidney |
| Transporter | SLC22A2 (OCT2) |
| Ki (µM) | 0.710 |
| fuinc (Ki) | 1.000 |
| Transporter | ABCB1 (P-gp/MDR1) |
| Ki (µM) | 10.000 |
| fuinc (Ki) | 1.000 |

# SM 5: Simulation designs

The simulation design information for each modeling stage was summarized below. In all simulations, fedratinib and other compounds were administered via the oral route in the fasted state unless indicated differently. In the table below, HV and Cancer refer to “Sim-Healthy Volunteers.lbrz” and “Sim-Cancer.lbrz” Simcyp® population files used in the corresponding simulations, respectively.

**Table 4: Summary of Simulation Design Information**

| **Modeling stage** | **Clinical data** | **Dosing regimen** | **Formulation** | **Dosing time** | **Overall period (day)** | **Population** | **No. of virtual trials** | **No. of subjects per trial** | **Age range (year)** | **Female portion** |
| --- | --- | --- | --- | --- | --- | --- | --- | --- | --- | --- |
| Development | TDU12620 BDR12462 FED12258 ALI13451 INT12893 | Fedratinib 500 mg once | Capsule | Day 1, 0 h | 7 | HV | 1  10 | 1  10 | 20  19-51 | 0 |
| Verification / modification | ARD11936 | Fedratinib 300 mg QD | Capsule | Day 1-29, 0h | 29 | Cancer  HV | 10 | 10 | 36-71  36-65 | 0.6 |
|  |  | Fedratinib 400 mg QD | Capsule | Day 1-29, 0h | 29 | Cancer  HV | 10 | 10 | 47-81  47-65 | 0.4 |
|  |  | Fedratinib 500 mg QD | Capsule | Day 1-29, 0h | 29 | Cancer or HV | 10 | 11 | 44-83  44-65 | 0.455 |
|  | TDU12620 INT12893 | Fedratinib 300 mg once | Capsule | Day 1, 0 h | 7 | HV | 10 | 10 | 19-51 | 0 |
|  | INT12893 | Fedratinib 300 mg once +  Ketoconazole 200 mg BID | Capsule  Tablet | Day 6, 0h  Day 1-14, 0h | 14 | HV | 10 | 6 | 22-45 | 0 |
|  | INT12497 | Midazolam 2 mg once | Solution | Day 1, 0 h | 15 | Cancer  HV | 10 | 16 | 50-82 or 50-65 | 0.375 |
|  |  | Omeprazole 20 mg once | Capsule | Day 1, 0 h | 15 | Cancer  HV | 10 | 16 | 50-82 or 50-65 | 0.375 |
|  |  | Metoprolol 100 mg once | Tablet | Day 1, 0 h | 15 | Cancer  HV | 10 | 16 | 50-82 or 50-65 | 0.375 |
|  |  | Fedratinib 500 mg QD + Midazolam 2 mg once + Omeprazole 20 mg once + Metoprolol 100 mg once | Capsule Solution Capsule Tablet | Day 1-15, 0h Day 15, 0h Day 15, 0h Day 15, 0h | 15 | Cancer  HV | 10 | 13 | 50-82 or 50-65 | 0.375 |

**Table 4: Summary of Simulation Design Information (Continued)**

| **Modeling stage** | **Clinical data** | **Dosing regimen** | **Formulation** | **Dosing time** | **Overall period (day)** | **Population ^a^)** | **No. of virtual trials** | **No. of subjects per trial** | **Age range (year)** | **Female portion (%)** |
| --- | --- | --- | --- | --- | --- | --- | --- | --- | --- | --- |
| Application – metabolic DDI (victim effect) | N/A | Fedratinib 400 mg once +  Ketoconazole 200 mg BID or Ketoconazole 400 mg QD or Ritonavir 100 mg BID or Itraconazole 200 mg QD (200 mg BID Day 1) or Clarithromycin 250 mg BID or Fluconazole 200 mg QD or Fluconazole 400 mg QD or Erythromycin 500 mg TID or Diltiazem 120 mg BID or Fluvoxamine 100 mg BID or Quinidine 200 mg QD | Capsule  Default | Day 6, 0h  Day 1-20, 0h | 20 | HV  Cancer | 10 | 6 | 22-45  36-83 | 0  0.5 |
|  | N/A | Fedratinib 400 mg QD +  Ketoconazole 200 mg BID or Ketoconazole 400 mg QD or Ritonavir 100 mg BID or Itraconazole 200 mg QD (200 mg BID Day 1) or Clarithromycin 250 mg BID or Fluconazole 200 mg QD or Fluconazole 400 mg QD or Erythromycin 500 mg TID or Diltiazem 120 mg BID or Fluvoxamine 100 mg BID or Quinidine 200 mg QD | Capsule  Default | Day 6-20, 0h  Day 1-20, 0h | 20 | HV  Cancer | 10 | 6 | 22-45  36-83 | 0  0.5 |
|  | N/A | Fedratinib 400 mg once +  Efavirenz 600 mg QD or Rifampicin 600 mg QD | Capsule  Default | Day 10, 0h  Day 1-25, 0h | 25 | HV  Cancer | 10 | 6 | 22-45  36-83 | 0  0.5 |
|  | N/A | Fedratinib 400 mg QD +  Efavirenz 600 mg QD or Rifampicin 600 mg QD | Capsule  Default | Day 10-25, 0h  Day 1-25, 0h | 25 | HV  Cancer | 10 | 6 | 22-45  36-83 | 0  0.5 |

**Table 4: Summary of Simulation Design Information (Continued)**

| **Modeling stage** | **Clinical data** | **Dosing regimen** | **Formulation** | **Dosing time** | **Overall period (day)** | **Population ^a^)** | **No. of virtual trials** | **No. of subjects per trial** | **Age range (year)** | **Female portion (%)** |
| --- | --- | --- | --- | --- | --- | --- | --- | --- | --- | --- |
| Application – metabolic DDI (perpetrator effect) | N/A | Fedratinib 400 mg QD +  Repaglinide 0.25 mg once or Warfarin 15 mg once | Capsule  Default | Day 1-25, 0h  Day 16, 0h | 25 | HV  Cancer | 10 | 10 | 19-51  50-82 | 0  0.375 |
|  | N/A | Fedratinib 400 mg QD +  Repaglinide 0.25 mg once or Warfarin 15 mg once | Capsule  Default | Day 1-25, 0h  Day 16, 0h | 25 | HV  Cancer | 10 | 10 | 19-51  50-82 | 0  0.375 |

BID = twice a day; DDI = drug-drug interaction; HV = healthy volunteers; N/A = not applicable; TID = three times a day; QD = once a day.

# SM 6: Summary of Model-Simulated PK Parameters in Myelofibrosis (MF) Patients Following Multiple Doses of Fedratinib

Table 5: Summary of Model-simulated and Clinically-observed Exposure Parameters in Healthy Subjects and MF Patients Following Single and Repeated Doses of Fedratinib Only

| **Clinical Scenario** | **Virtual Subjects** | **Cmax (ng/mL)** | | | **AUC (ng/mL·h)** | | |
| --- | --- | --- | --- | --- | --- | --- | --- |
|  |  | **Observation** | **Prediction** | **PE (%)** | **Observation** | **Prediction** | **PE (%)** |
| 300 mg single dose in healthy subjects | Healthy | 521 (189)  [497] | 656 (404) [551] | 11 | 6730 (1990) [6530] | 9950 (5770)  [8560] | 31 |
| 500 mg single dose in healthy subjects | Healthy | 720 (243) [683] | 1130 (693) [950] | 39 | 13900 (5240) [13200] | 18800 (11100) [16100] | 22 |
| 300 mg repeated doses in MF patients (Day 1) | Healthy | 1380 (1060) [1070] | 707 (465) [584] | -45 | 9460 (7770) [7020] | 7390 (3650) [6530] | -7 |
|  | Cancer |  | 984 (574) [832] | -22 |  | 11100 (4800) [10100] | 44 |
| 300 mg repeated doses in MF patients (Steady state) | Healthy | 1800 (1120) [1530] | 1450 (830) [1240] | -19 | 25300 (14100) [22400] | 18600 (10100) [15900] | -29 |
|  | Cancer |  | 2240 (1230)  [1930] | 26 |  | 33600 (17200) [29300] | 31 |
| 400 mg repeated doses in MF patients (Day 1) | Healthy | 1430 (754) [1290] | 1020 (696) [834] | -35 | 9610 (3450) [9130] | 11100 (5980] [9760] | 7 |
|  | Cancer |  | 1390 (837) [1170] | -9 |  | 16200 (7150) [14700] | 61 |
| 400 mg repeated doses in MF patients (Steady state) | Healthy | 2020 (1250) [1800] | 2130 (1320) [1800] | 0 | 29100 (13500) [26900) | 29000 (18200) [24200] | -10 |
|  | Cancer |  | 3250 (1780) [2810] | 56 |  | 50800 (25700) [44300] | 65 |
| 500 mg repeated doses in MF patients (Day 1) | Healthy | 1420 (710) [1270] | 1320 (845) [1100] | -13 | 11300 (6060)  [9980] | 14700 (7000) [13300] | 33 |
|  | Cancer |  | 1820 (1240) [1490] | 17 |  | 21400 (10700) [19100] | 91 |
| 500 mg repeated doses in MF patients (Steady state) | Healthy | 2830 (1370) [2540] | 2800 (1510) [2440] | -4 | 44500 (24900) [38700] | 37900 (20100) [33200] | -14 |
|  | Cancer |  | 4280 (2540) [3650] | 44 |  | 66900 (36300) [57900] | 50 |

AUC refers to AUC_inf_ for the single dose scenarios and AUCτ for the repeated QD dose scenarios. The number of subjects (n) = 6 in TDU12620, n = 17 in INT12894, n = 10-11 in ARD11936, and n = 100 in virtual trial simulations, respectively. All model simulations listed in the table were simulated using the default “Healthy Volunteers” and “Cancer” population files provided in Simcyp® (V17R1). The prediction error is calculated using PE = (GM_prediction_ – GM_observation_)/GM_observation_ ×100%, where GM refers to geometric mean. SD refers to standard deviation.

# SM 7: Model-simulated and Clinically Observed Mean Time-concentration Profiles of Fedratinib or CYP Substrates in Presence or Absence of Interaction

| 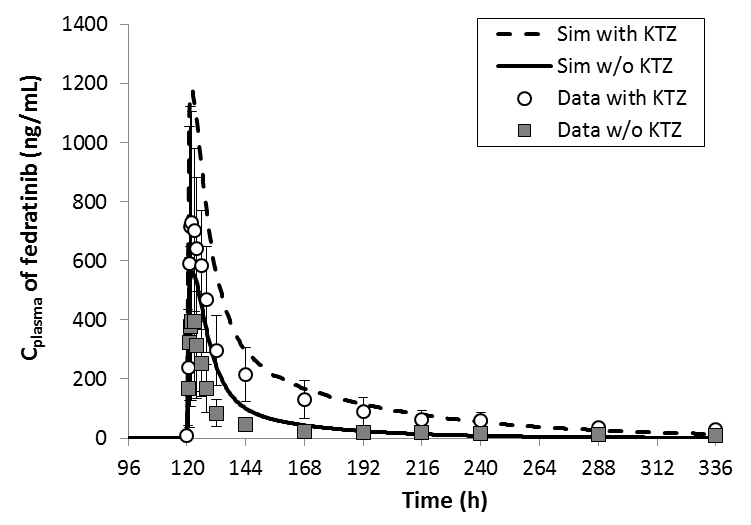  A | 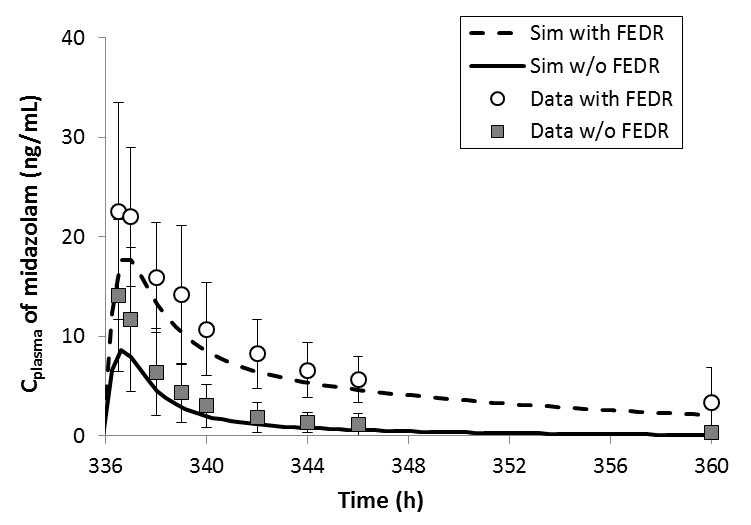  B |
| --- | --- |
| 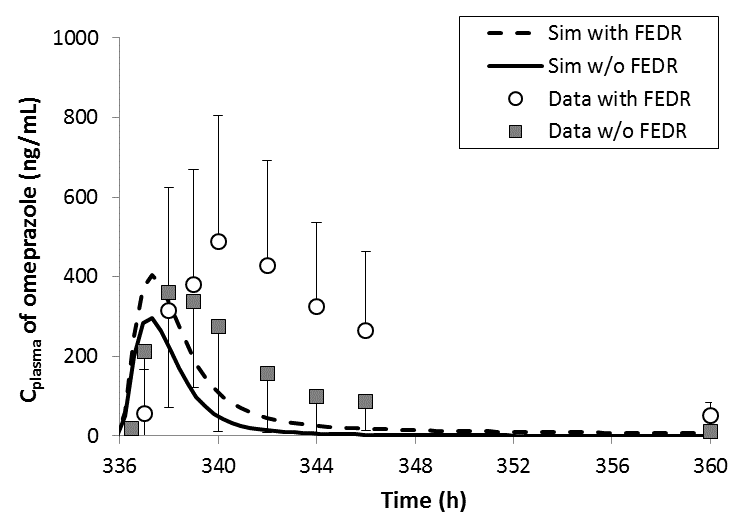  C | 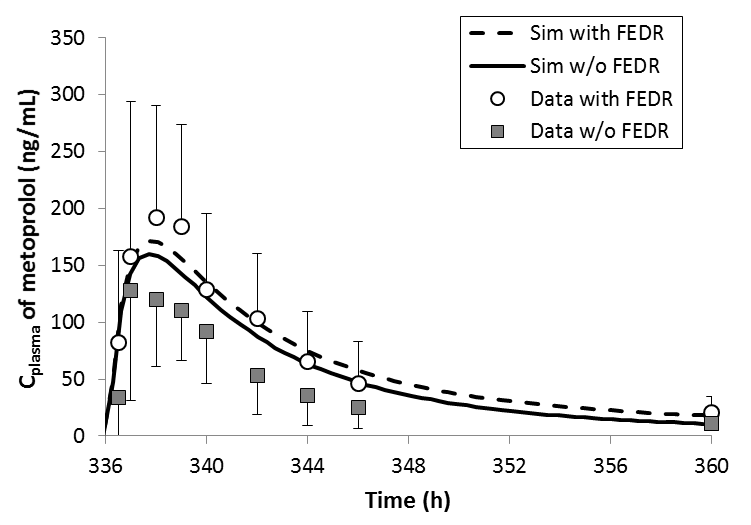  D |
| Figure 3: Model-simulated and Clinically-observed Mean Concentration-Time Profiles of Fedratinib or CYP Substrates in Presence or Absence of Interaction. Subplot A: C_plasma_ of fedratinib following a single dose of 200 mg fedratinib in presence or absence of 200 mg BID ketoconazole; Subplot B: C_plasma_ of midazolam following a single dose of 2 mg midazolam in presence or absence of 500 mg QD fedratinib; Subplot C: C_plasma_ of omeprazole following a single dose of 20 mg omeprazole in presence or absence of 500 mg QD fedratinib; Subplot D: C_plasma_ of metoprolol following a single dose of 100 mg metoprolol in presence or absence of 500 mg QD fedratinib. The model-predicted time-concentration profiles without and with the drug interaction were plotted as solid and dashed curves, respectively. The experimentally observed mean PK data without and with the drug interaction were plotted as solid and open symbols, respectively. Error bars indicate standard deviations. | |

# SM 8: Parameter sensitivity analysis

## Manual sensitivity analysis on fm

The parameter sensitivity analysis was conducted to understand impacts of fm_CYP3A4_ and fm of additional HLM (fm_HLM,add_) on the accumulation ratio of exposure (AUC) following the repeated doses of 500 mg QD fedratinib by using the Simcyp Cancer population files. As shown in Table 6, agreement between the predicted and observed accumulation ratio of exposure following 500 mg QD doses of fedratinib was improved by incorporating the additional HLM clearance into the CYP3A4 mediated clearance.

Table 6: Summary of Parameter Sensitivity Analysis on Fraction of Metabolism in MF Patients Following Repeated Doses (QD) of 500 mg Fedratinib

|  | CYP3A4 | | Additional HLM | | Day 1 | Steady state | Accumulation Ratio |
| --- | --- | --- | --- | --- | --- | --- | --- |
|  | fm | Hep Met CL (%) | fm | Hep Met CL (%) | AUC_0-24h_ (ng/mL·h) | AUC_τ_ (ng/mL·h) |  |
| Simulation “Cancer” 500 mg QD | 0.62 | 81 | 0 | 0 | 19100 | 57900 | 3.0 |
|  | 0.55 | 72 | 0.07 | 9 | 19000 | 52100 | 2.7 |
|  | 0.48 | 63 | 0.14 | 18 | 19000 | 47800 | 2.5 |
| Study ARD11936  500 mg QD |  | - |  | - | 9980 | 38700 | 3.9 |

## PSA on KTZ DDI

Further parameter sensitivity analysis (PSA) was conducted to investigate impacts of various parameters on model-predicted DDI between fedratinib (300 mg once) and ketoconazole (200 mg BID) following the design of Study [INT12893](#references). As shown in Figure 4, the model-predicted AUC ratio, which is defined as (AUC_inf_ with inh)/(AUC_inf_ w/o inh), was found to decrease from ~3.1 to ~2.8 when dose increases from 300 to 500 mg, increase from ~1 to ~8 when fm_CYP3A4_ increases from 10% to 90%, increase from ~2.6 to ~3.1 when fu_Gut_ increases from 0.1 to 1, and decrease from ~6 to ~3 as Q_Gut_ increases from 1 to 12 L/h. In summary, the model-predicted DDI extent (in terms of AUC ratio) is sensitive to fm_CYP3A4_, but not to dose, fu_Gut_, and Q_Gut_ around the model parameter values.

| 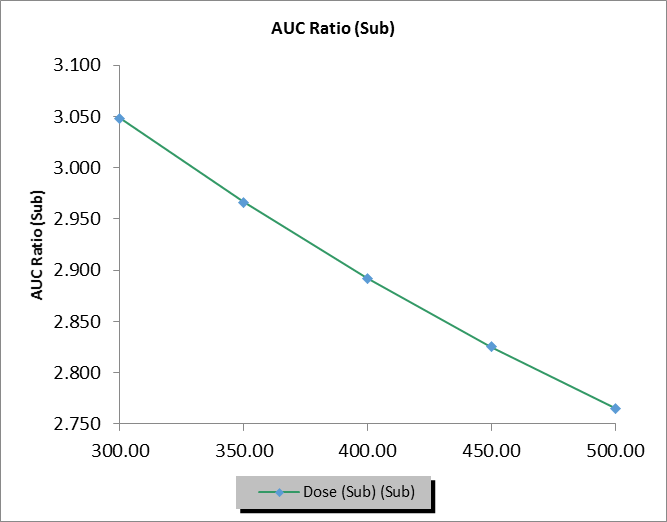  Dose: 300 to 500 mg | 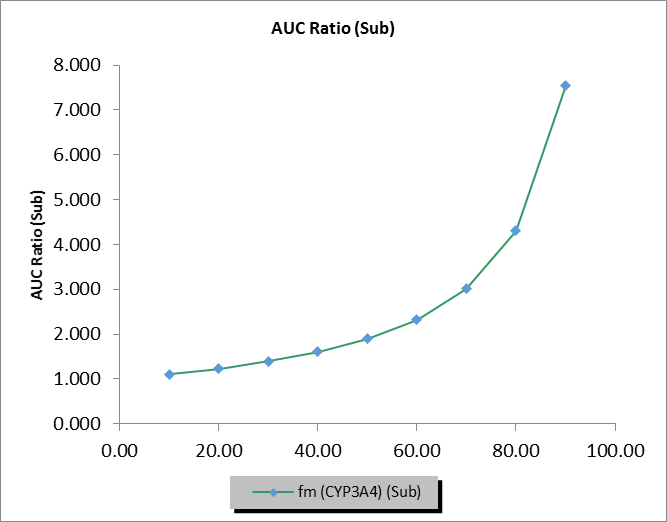  fm_CYP3A4_: 10% to 90% |
| --- | --- |
| 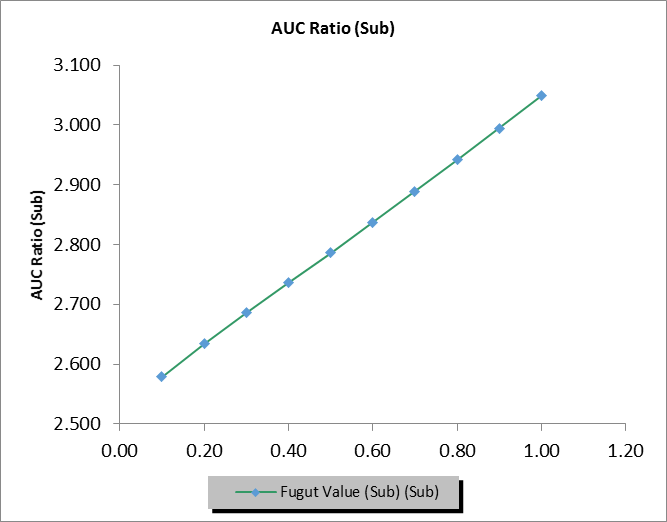  fu_Gut_: 0.1 to 1 | 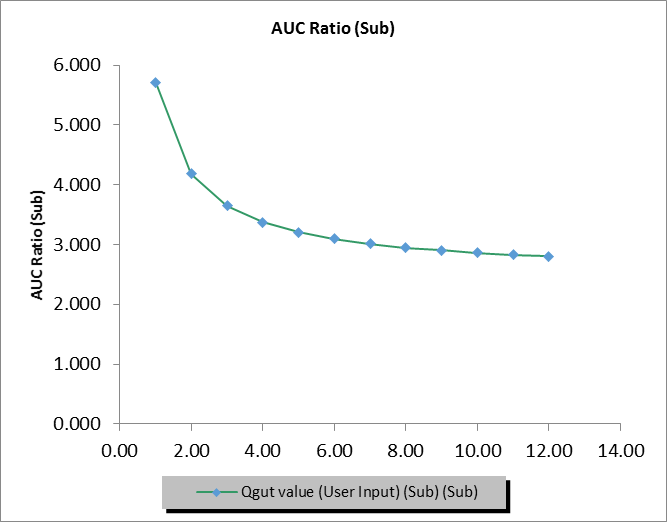  Q_Gut_: 1 to 12 L/h |

**Figure 4: Parameter Sensitivity Analysis of Q_Gut_ on Drug-drug Interaction Magnitude between Fedratinib (300 mg once) and Ketoconazole (200 mg BID)**

## PSA on repaglinide DDI

The parameter sensitivity analyses for repaglinide were conducted using the default “Healthy Volunteers” population given 400 mg QD doses of fedratinib (as the perpetrator) and a single 0.25 mg dose of repaglinide (as the victim). A minimal 10-fold range was used in the sensitivity analyses with a larger range used for time-dependent inhibition and inhibition parameters. As summarized in Figure 5, the sensitivity analyses suggest minor impacts of fedratinib CYP2C8 Ki, CYP3A4 Ki, and OATP1B1 Ki on AUC ratio over the tested parameter ranges. Since the repaglinide is not characterized as the OATP1B3 transporter substrate in Simcyp® (Version 17), the changes of fedratinib OATP1B3 Ki do not impact the simulation results. The sensitivity analyses on the time-dependent inhibition and induction parameters suggest that AUC ratio decreases from ~1.5 to ~1.3 when fedratinib CYP3A4 K_app_ increases from 0.1 to 2 μM, increases from ~1.1 to ~1.5 when fedratinib CYP3A4 k_inact_ increases from 1 to 50 1/h, decreases from ~1.5 to ~1.1 when fedratinib CYP3A4 Ind_max_ increases from 2 to 200, and increases from ~0.5 to ~1 when fedratinib CYP3A4 Ind_C50_ increases from 0.1 to 1 μM while increases from ~1 to ~1.5 with further increases in fedratinib CYP3A4 Ind_C50_ from 1 to 60 μM. In summary, the model-predicted DDI extent (in terms of AUC ratio) is insensitive to the fedratinib interaction parameters, including CYP2C8 Ki, CYP3A4 Ki, CYP3A4 K_app_, CYP3A4 k_inact_, CYP3A4 Ind_max_, CYP3A4 Ind_C50_, OATP1B1 Ki, OATP1B3 Ki, around the model parameter values.

| 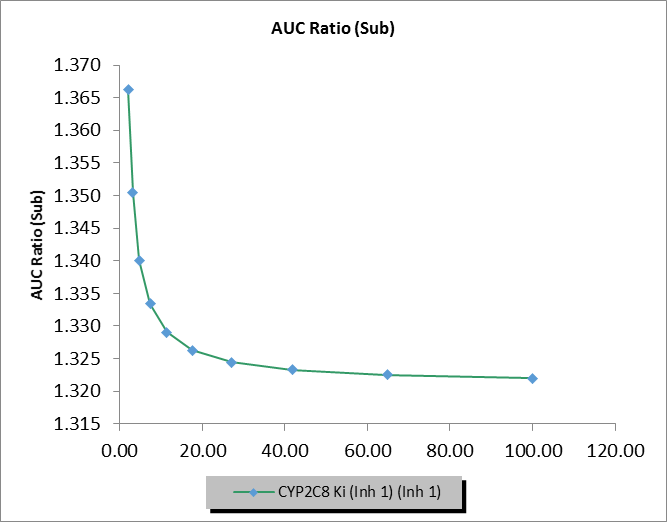  Fedratinib CYP2C8 Ki (2 to 100 μM) | 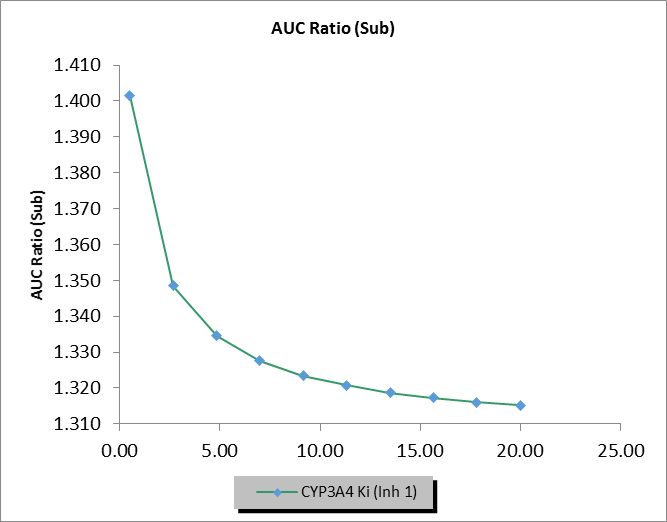  Fedratinib CYP3A4 Ki (0.5 to 20 μM) |
| --- | --- |
| 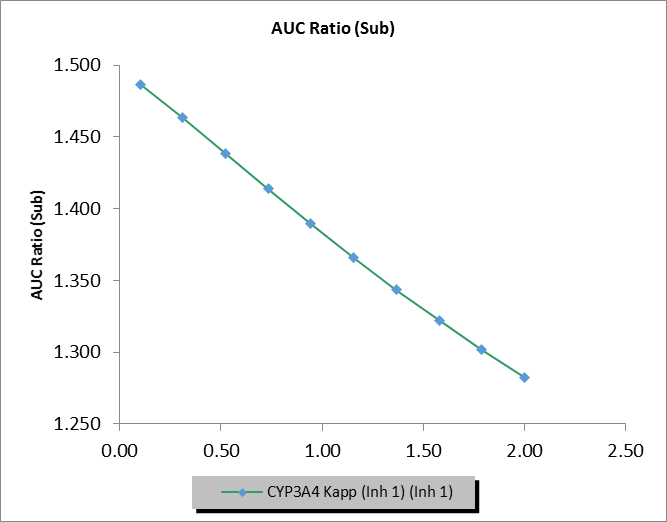  Fedratinib CYP3A4 K_app_ (0.1 to 2 μM) | 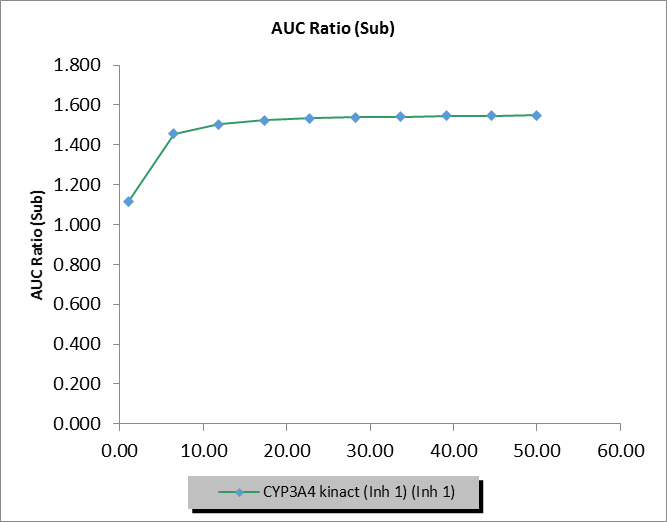  Fedratinib CYP3A4 k_inact_ (1 to 50 1/h) |
| 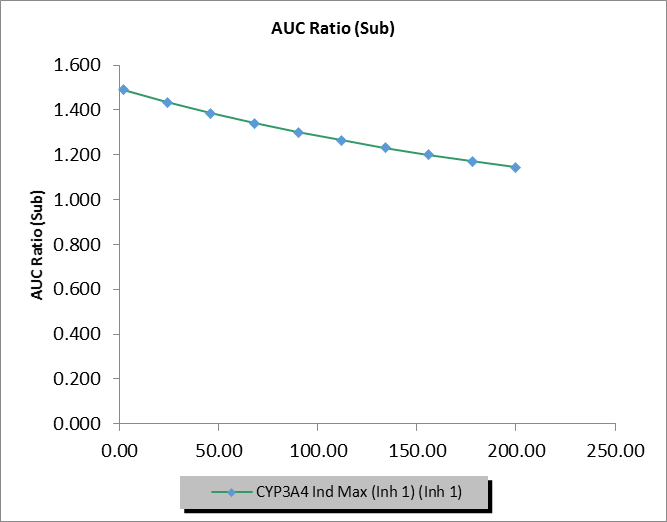  Fedratinib CYP3A4 Ind_max_ (2 to 200) | 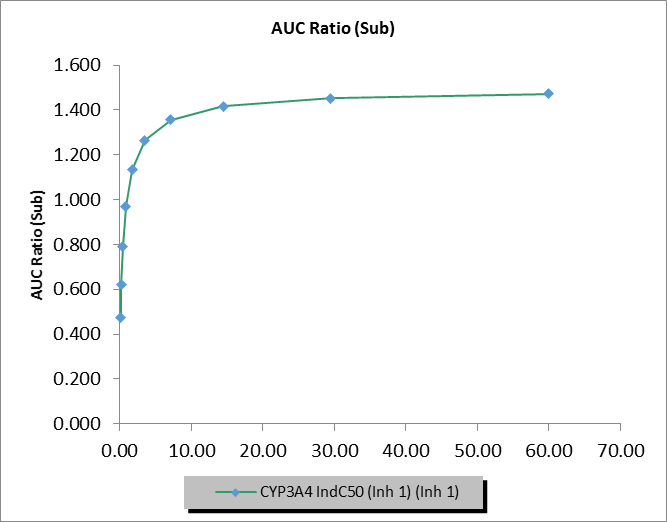  Fedratinib CYP3A4 Ind_C50_ (0.1 to 60 μM) |
| 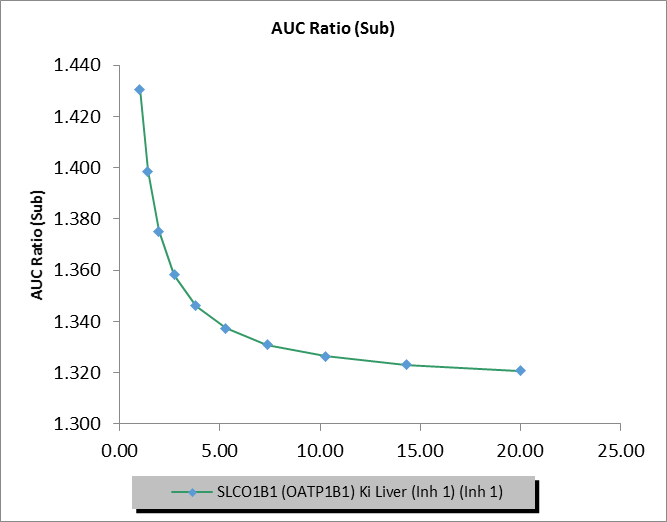  Fedratinib OATP1B1 Ki (1 to 20 μM) | 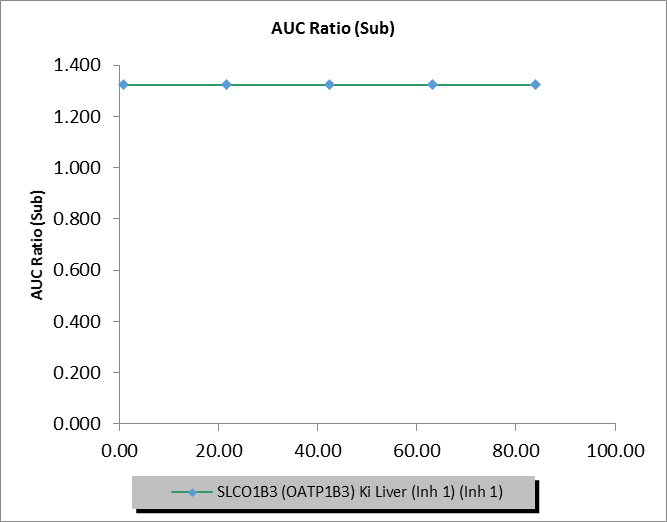  Fedratinib OATP1B3 Ki (0.84 to 84 μM) |

**Figure 5: Sensitivity Analyses of Fedratinib Interaction Parameters on Drug-Drug Interaction Magnitude as AUC Ratio with Repaglinide as the Victim**

## PSA on warfarin DDI

The parameter sensitivity analysis for warfarin was conducted using the default “Healthy Volunteers” population given 400 mg QD doses of fedratinib (as the perpetrator) and a single 15 mg dose of warfarin (as the victim). As summarized in Figure 6 , the sensitivity analyses suggest minor impacts of fedratinib CYP2C9 Ki on AUC ratio over the tested parameter range.

| 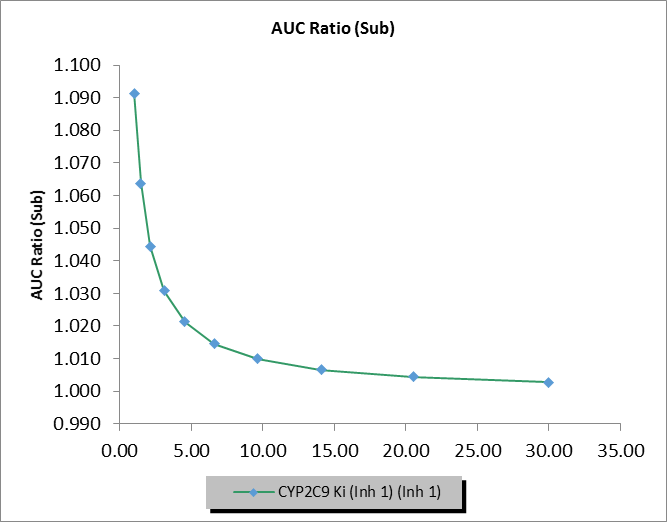  Fedratinib CYP2C9 Ki (1 to 30 μM) |
| --- |

**Figure 6: Sensitivity Analyses of Fedratinib Interaction Parameters on Drug-Drug Interaction Magnitude as AUC Ratio with Warfarin as the Victim**

In summary, the sensitivity analysis over a 10-fold or larger range of parameters shows no notable impact on the extent of interaction predicted for repaglinide and warfarin.

# SM 9: Model-Predicted Metabolism-mediated Drug-drug Interactions with Fedratinib as the Victim

The class of each modulator along with victim and perpetrator dose regimen was summarized in Table 7.

Table 7: List of Modulators used for the Metabolism-mediated DDI Assessment with Fedratinib as the Victim

| Victim | Perpetrator | Perpetrator class |
| --- | --- | --- |
| Fedratinib (400 mg once) | Ketoconazole (200 mg BID) | CYP3A4 strong inhibitor |
|  | Ketoconazole (400 mg QD) | CYP3A4 strong inhibitor |
|  | Ritonavir (100 mg BID) | CYP3A4 strong inhibitor |
|  | Itraconazole (200 mg BID at Day 1 + 200 mg QD from Day 2) | CYP3A4 strong inhibitor |
|  | Clarithromycin (250 mg BID) | CYP3A4 strong inhibitor |
|  | Fluconazole (200 mg QD) | CYP3A4 moderate inhibitor  CYP2C19 strong inhibitor |
|  | Fluconazole (400 mg QD) | CYP3A4 moderate inhibitor  CYP2C19 strong inhibitor |
|  | Erythromycin (500 mg TID) | CYP3A4 moderate inhibitor |
|  | Diltiazem (120 mg BID) | CYP3A4 moderate inhibitor |
|  | Fluvoxamine (100 mg BID) | CYP3A4 moderate inhibitor  CYP2C19 strong inhibitor |
|  | Quinidine (200 mg QD) | CYP3A4 weak inhibitor  CYP2D6 strong inhibitor |
|  | Efavirenz (600 mg QD) | CYP3A4 moderate inducer |
|  | Rifampicin (600 mg QD) | CYP3A4 strong inducer |

Table 8: Model-predicted PK and Drug-drug Interaction Magnitudes Following a Single Dose (400 mg Once) of Fedratinib (as the Victim) and Repeated Doses of CYP3A4 Modulators in Healthy Subjects

| Perpetrator (dosing regimen) | Parameter | C_max_ w/o inh (ng/mL) | C_max_ with inh (ng/mL) | C_max_ Ratio | AUC_inf_ w/o inh (ng/mL·h) | AUC_inf_ with inh (ng/mL·h) | AUC ratio |
| --- | --- | --- | --- | --- | --- | --- | --- |
| Ketoconazole (200 mg BID) | Mean | 875 | 1850 | 2.12 | 14600 | 42400 | 3.27 |
|  | SD | 636 | 1370 | 0.528 | 9720 | 21400 | 1.52 |
|  | GeoMean | 719 | 1480 | 2.05 | 12200 | 36800 | 3.02 |
|  | 90% CI lower | 632 | 1280 | 1.95 | 10700 | 32500 | 2.78 |
|  | 90% CI upper | 819 | 1710 | 2.17 | 13900 | 41700 | 3.28 |
| Ketoconazole (400 mg QD) | Mean | 875 | 1880 | 2.15 | 14600 | 40600 | 3.1 |
|  | SD | 636 | 1390 | 0.546 | 9720 | 21000 | 1.39 |
|  | GeoMean | 719 | 1500 | 2.08 | 12200 | 35100 | 2.88 |
|  | 90% CI lower | 632 | 1300 | 1.97 | 10700 | 31000 | 2.65 |
|  | 90% CI upper | 819 | 1730 | 2.2 | 13900 | 39800 | 3.12 |
| Ritonavir  (100 mg BID) | Mean | 885 | 1730 | 1.96 | 14800 | 40700 | 3.04 |
|  | SD | 662 | 1270 | 0.419 | 9890 | 21200 | 1.36 |
|  | GeoMean | 722 | 1390 | 1.92 | 12300 | 34900 | 2.84 |
|  | 90% CI lower | 634 | 1200 | 1.83 | 10800 | 30600 | 2.63 |
|  | 90% CI upper | 824 | 1600 | 2.01 | 14000 | 39700 | 3.06 |
| Itraconazole (200 mg BID at Day 1 + 200 mg QD from Day 2) | Mean | 886 | 2110 | 2.33 | 14500 | 45400 | 3.45 |
|  | SD | 461 | 1440 | 0.831 | 9150 | 24000 | 1.53 |
|  | GeoMean | 769 | 1700 | 2.21 | 12200 | 39000 | 3.19 |
|  | 90% CI lower | 682 | 1470 | 2.06 | 10700 | 34300 | 2.93 |
|  | 90% CI upper | 867 | 1970 | 2.37 | 13900 | 44300 | 3.46 |
| Clarithromycin (250 mg BID) | Mean | 885 | 1380 | 1.51 | 14800 | 25000 | 1.72 |
|  | SD | 662 | 1140 | 0.245 | 9890 | 15700 | 0.441 |
|  | GeoMean | 722 | 1080 | 1.49 | 12300 | 20600 | 1.67 |
|  | 90% CI lower | 634 | 934 | 1.44 | 10800 | 17900 | 1.6 |
|  | 90% CI upper | 824 | 1250 | 1.55 | 14000 | 23700 | 1.76 |
| Fluconazole (200 mg QD) | Mean | 853 | 1770 | 2.06 | 14700 | 44600 | 3.47 |
|  | SD | 611 | 1300 | 0.579 | 10000 | 21400 | 1.06 |
|  | GeoMean | 697 | 1390 | 1.99 | 12100 | 40300 | 3.34 |
|  | 90% CI lower | 608 | 1190 | 1.89 | 10500 | 36500 | 3.15 |
|  | 90% CI upper | 798 | 1620 | 2.11 | 13800 | 44500 | 3.54 |
| Fluconazole (400 mg QD) | Mean | 853 | 2050 | 2.39 | 14700 | 57000 | 4.64 |
|  | SD | 611 | 1530 | 0.822 | 10000 | 23400 | 1.80 |
|  | GeoMean | 697 | 1580 | 2.27 | 12100 | 52700 | 4.37 |
|  | 90% CI lower | 608 | 1350 | 2.12 | 10500 | 48300 | 4.06 |
|  | 90% CI upper | 798 | 1860 | 2.43 | 13800 | 57500 | 4.71 |
| Erythromycin (500 mg TID) | Mean | 875 | 1410 | 1.61 | 14600 | 26200 | 1.95 |
|  | SD | 636 | 1030 | 0.312 | 9720 | 14400 | 0.834 |
|  | GeoMean | 719 | 1140 | 1.58 | 12200 | 22500 | 1.85 |
|  | 90% CI lower | 632 | 990 | 1.52 | 10700 | 19900 | 1.73 |
|  | 90% CI upper | 819 | 1310 | 1.65 | 13900 | 25600 | 1.97 |
| Diltiazem (120 mg BID) | Mean | 879 | 1400 | 1.55 | 14300 | 22900 | 1.65 |
|  | SD | 466 | 898 | 0.31 | 8950 | 13200 | 0.39 |
|  | GeoMean | 761 | 1160 | 1.52 | 12000 | 19400 | 1.62 |
|  | 90% CI lower | 675 | 1010 | 1.46 | 10500 | 17100 | 1.55 |
|  | 90% CI upper | 858 | 1330 | 1.58 | 13700 | 22100 | 1.69 |
| Fluvoxamine (100 mg BID) | Mean | 875 | 1200 | 1.37 | 14600 | 24100 | 1.72 |
|  | SD | 636 | 876 | 0.22 | 9720 | 15100 | 0.42 |
|  | GeoMean | 719 | 979 | 1.36 | 12200 | 20600 | 1.69 |
|  | 90% CI lower | 632 | 856 | 1.32 | 10700 | 18200 | 1.61 |
|  | 90% CI upper | 819 | 1120 | 1.4 | 13900 | 23300 | 1.76 |
| Quinidine (200 mg QD) | Mean | 878 | 1090 | 1.22 | 14400 | 17100 | 1.18 |
|  | SD | 455 | 602 | 0.13 | 9270 | 11400 | 0.07 |
|  | GeoMean | 763 | 925 | 1.21 | 12000 | 14200 | 1.18 |
|  | 90% CI lower | 677 | 814 | 1.19 | 10500 | 12400 | 1.16 |
|  | 90% CI upper | 860 | 1050 | 1.24 | 13700 | 16200 | 1.19 |
| Efavirenz (600 mg QD) | Mean | 867 | 467 | 0.556 | 14200 | 6150 | 0.434 |
|  | SD | 639 | 376 | 0.169 | 9090 | 4990 | 0.167 |
|  | GeoMean | 710 | 375 | 0.528 | 12000 | 4780 | 0.400 |
|  | 90% CI lower | 623 | 328 | 0.491 | 10500 | 4100 | 0.365 |
|  | 90% CI upper | 809 | 429 | 0.568 | 13600 | 5570 | 0.438 |
| Rifampicin (600 mg QD) | Mean | 853 | 229 | 0.285 | 14700 | 2560 | 0.174 |
|  | SD | 611 | 181 | 0.143 | 10000 | 2180 | 0.087 |
|  | GeoMean | 697 | 172 | 0.247 | 12100 | 1860 | 0.154 |
|  | 90% CI lower | 608 | 145 | 0.218 | 10500 | 1550 | 0.138 |
|  | 90% CI upper | 798 | 204 | 0.28 | 13800 | 2230 | 0.172 |

AUC Ratio = (AUC_inf_ with inh)/(AUC_inf_ w/o inh); C_max_ ratio = (C_max_ with inh)/(C_max_ w/o inh); CI = confidence interval; GeoMean = geometric mean; inh = inhibition; N = the number of subjects; N/A = not available; w/o = without.

Table 9: Model-predicted PK and Drug-drug Interaction Magnitudes Following a Single Dose (400 mg Once) of Fedratinib (as the Victim) and Repeated Doses of CYP3A4 Modulators in Cancer Patients

| Perpetrator (dosing regimen) | Parameter | C_max_ w/o inh (ng/mL) | C_max_ with inh (ng/mL) | C_max_ Ratio | AUC_inf_ w/o inh (ng/mL·h) | AUC_inf_ with inh (ng/mL·h) | AUC ratio |
| --- | --- | --- | --- | --- | --- | --- | --- |
| Ketoconazole (200 mg BID) | Mean | 1370 | 2270 | 1.66 | 35200 | 81600 | 2.6 |
|  | SD | 916 | 1530 | 0.343 | 21400 | 36000 | 0.943 |
|  | GeoMean | 1140 | 1860 | 1.63 | 29600 | 73200 | 2.47 |
|  | 90% CI lower | 1000 | 1620 | 1.56 | 26000 | 65700 | 2.31 |
|  | 90% CI upper | 1300 | 2140 | 1.7 | 33800 | 81500 | 2.64 |
| Ketoconazole (400 mg QD) | Mean | 1370 | 2290 | 1.68 | 35200 | 77300 | 2.44 |
|  | SD | 916 | 1540 | 0.352 | 21400 | 35300 | 0.847 |
|  | GeoMean | 1140 | 1870 | 1.64 | 29600 | 69000 | 2.33 |
|  | 90% CI lower | 1000 | 1630 | 1.57 | 26000 | 61800 | 2.18 |
|  | 90% CI upper | 1300 | 2160 | 1.72 | 33800 | 77000 | 2.48 |
| Ritonavir (100 mg BID) | Mean | 1380 | 2160 | 1.58 | 35200 | 81600 | 2.62 |
|  | SD | 950 | 1420 | 0.284 | 22000 | 35500 | 0.988 |
|  | GeoMean | 1140 | 1780 | 1.56 | 29600 | 73300 | 2.48 |
|  | 90% CI lower | 1000 | 1550 | 1.5 | 26000 | 65800 | 2.31 |
|  | 90% CI upper | 1300 | 2040 | 1.61 | 33700 | 81600 | 2.65 |
| Itraconazole (200 mg BID at Day 1 + 200 mg QD from Day 2) | Mean | 1420 | 2600 | 1.79 | 35300 | 88200 | 2.83 |
|  | SD | 770 | 1690 | 0.51 | 21500 | 40600 | 1.15 |
|  | GeoMean | 1230 | 2130 | 1.73 | 29800 | 78900 | 2.65 |
|  | 90% CI lower | 1090 | 1850 | 1.64 | 26100 | 70800 | 2.46 |
|  | 90% CI upper | 1390 | 2450 | 1.83 | 33900 | 88000 | 2.86 |
| Clarithromycin (250 mg BID) | Mean | 1380 | 1950 | 1.39 | 35200 | 59300 | 1.74 |
|  | SD | 950 | 1390 | 0.19 | 22000 | 33200 | 0.435 |
|  | GeoMean | 1140 | 1580 | 1.38 | 29600 | 50300 | 1.7 |
|  | 90% CI lower | 1000 | 1370 | 1.34 | 26000 | 44100 | 1.63 |
|  | 90% CI upper | 1300 | 1820 | 1.42 | 33700 | 57400 | 1.78 |
| Fluconazole (200 mg QD) | Mean | 1390 | 2300 | 1.62 | 35200 | 84700 | 2.73 |
|  | SD | 969 | 1700 | 0.327 | 23000 | 38000 | 0.765 |
|  | GeoMean | 1100 | 1760 | 1.59 | 29100 | 76700 | 2.64 |
|  | 90% CI lower | 950 | 1490 | 1.53 | 25400 | 69500 | 2.49 |
|  | 90% CI upper | 1280 | 2070 | 1.66 | 33200 | 84700 | 2.79 |
| Fluconazole (400 mg QD) | Mean | 1390 | 2500 | 1.76 | 35200 | 100000 | 3.34 |
|  | SD | 969 | 1900 | 0.425 | 23000 | 40200 | 1.1 |
|  | GeoMean | 1100 | 1890 | 1.72 | 29100 | 92500 | 3.18 |
|  | 90% CI lower | 950 | 1600 | 1.63 | 25400 | 84600 | 2.97 |
|  | 90% CI upper | 1280 | 2240 | 1.8 | 33200 | 101000 | 3.4 |
| Erythromycin (500 mg TID) | Mean | 1370 | 1810 | 1.32 | 35200 | 51600 | 1.58 |
|  | SD | 916 | 1170 | 0.2 | 21400 | 25900 | 0.473 |
|  | GeoMean | 1140 | 1490 | 1.31 | 29600 | 45300 | 1.53 |
|  | 90% CI lower | 1000 | 1310 | 1.27 | 26000 | 40300 | 1.45 |
|  | 90% CI upper | 1300 | 1710 | 1.35 | 33800 | 50900 | 1.61 |
| Diltiazem (120 mg BID) | Mean | 1410 | 2020 | 1.39 | 34500 | 53800 | 1.62 |
|  | SD | 770 | 1320 | 0.264 | 20200 | 29000 | 0.324 |
|  | GeoMean | 1220 | 1670 | 1.37 | 29200 | 46300 | 1.59 |
|  | 90% CI lower | 1080 | 1450 | 1.32 | 25600 | 40900 | 1.53 |
|  | 90% CI upper | 1370 | 1910 | 1.42 | 33200 | 52400 | 1.65 |
| Fluvoxamine (100 mg BID) | Mean | 1370 | 1710 | 1.24 | 35200 | 53400 | 1.57 |
|  | SD | 916 | 1150 | 0.137 | 21400 | 30100 | 0.304 |
|  | GeoMean | 1140 | 1410 | 1.24 | 29600 | 45800 | 1.55 |
|  | 90% CI lower | 1000 | 1240 | 1.21 | 26000 | 40600 | 1.49 |
|  | 90% CI upper | 1300 | 1610 | 1.27 | 33800 | 51800 | 1.6 |
| Quinidine (200 mg QD) | Mean | 1420 | 1710 | 1.19 | 35100 | 42300 | 1.2 |
|  | SD | 772 | 975 | 0.107 | 22000 | 27100 | 0.071 |
|  | GeoMean | 1230 | 1460 | 1.19 | 29300 | 35100 | 1.2 |
|  | 90% CI lower | 1090 | 1280 | 1.16 | 25700 | 30700 | 1.18 |
|  | 90% CI upper | 1380 | 1650 | 1.21 | 33500 | 40200 | 1.21 |
| Efavirenz (600 mg QD) | Mean | 1360 | 860 | 0.629 | 34700 | 16200 | 0.439 |
|  | SD | 930 | 690 | 0.153 | 21600 | 13700 | 0.15 |
|  | GeoMean | 1130 | 684 | 0.607 | 29100 | 12000 | 0.413 |
|  | 90% CI lower | 989 | 595 | 0.571 | 25600 | 10200 | 0.382 |
|  | 90% CI upper | 1280 | 787 | 0.646 | 33200 | 14300 | 0.447 |
| Rifampicin (600 mg QD) | Mean | 1390 | 556 | 0.414 | 35200 | 8210 | 0.22 |
|  | SD | 969 | 443 | 0.168 | 23000 | 7540 | 0.101 |
|  | GeoMean | 1100 | 412 | 0.373 | 29100 | 5690 | 0.196 |
|  | 90% CI lower | 950 | 346 | 0.335 | 25400 | 4700 | 0.175 |
|  | 90% CI upper | 1280 | 489 | 0.416 | 33200 | 6870 | 0.218 |

AUC Ratio = (AUC_inf_ with inh)/(AUC_inf_ w/o inh); C_max_ ratio = (C_max_ with inh)/(C_max_ w/o inh); CI = confidence interval; GeoMean = geometric mean; inh = inhibition; N = the number of subjects; N/A = not available; w/o = without.

Table 10: Model-predicted PK and Drug-drug Interaction Magnitudes Following Repeated Doses (400 mg QD) of Fedratinib (as the Victim) and Repeated Doses of CYP3A4 Modulators in Healthy Subjects

| Perpetrator (dosing regimen) | Parameter | C_max_ w/o inh (ng/mL) | C_max_ with inh (ng/mL) | C_max_ Ratio | AUC_τ_ w/o inh (ng/mL·h) | AUC_τ_ with inh (ng/mL·h) | AUC ratio |
| --- | --- | --- | --- | --- | --- | --- | --- |
| Ketoconazole (200 mg BID) | Mean | 1820 | 3150 | 1.84 | 24000 | 46900 | 2.13 |
|  | SD | 1260 | 1930 | 0.463 | 14500 | 23800 | 0.701 |
|  | GeoMean | 1510 | 2690 | 1.78 | 20100 | 40600 | 2.02 |
|  | 90% CI lower | 1330 | 2390 | 1.68 | 17500 | 35700 | 1.88 |
|  | 90% CI upper | 1720 | 3040 | 1.88 | 23100 | 46100 | 2.16 |
| Ketoconazole (400 mg QD) | Mean | 1820 | 3100 | 1.8 | 24000 | 45300 | 2.04 |
|  | SD | 1260 | 1940 | 0.436 | 14500 | 23300 | 0.65 |
|  | GeoMean | 1510 | 2640 | 1.74 | 20100 | 39100 | 1.95 |
|  | 90% CI lower | 1330 | 2340 | 1.66 | 17500 | 34500 | 1.82 |
|  | 90% CI upper | 1720 | 2980 | 1.84 | 23100 | 44400 | 2.08 |
| Ritonavir (100 mg BID) | Mean | 1870 | 2700 | 1.53 | 24700 | 39500 | 1.69 |
|  | SD | 1390 | 1710 | 0.223 | 15400 | 21100 | 0.299 |
|  | GeoMean | 1520 | 2300 | 1.51 | 20300 | 33800 | 1.67 |
|  | 90% CI lower | 1330 | 2040 | 1.46 | 17600 | 29700 | 1.60 |
|  | 90% CI upper | 1740 | 2600 | 1.56 | 23500 | 38600 | 1.73 |
| Itraconazole (200 mg BID at Day 1 + 200 mg QD from Day 2) | Mean | 1990 | 3530 | 1.92 | 24200 | 49700 | 2.28 |
|  | SD | 1300 | 1940 | 0.665 | 14400 | 25700 | 0.938 |
|  | GeoMean | 1650 | 3040 | 1.84 | 20000 | 42800 | 2.14 |
|  | 90% CI lower | 1440 | 2680 | 1.73 | 17400 | 37700 | 1.98 |
|  | 90% CI upper | 1890 | 3440 | 1.96 | 23100 | 48700 | 2.30 |
| Clarithromycin (250 mg BID) | Mean | 1870 | 2150 | 1.16 | 24700 | 28600 | 1.17 |
|  | SD | 1390 | 1550 | 0.060 | 15400 | 17000 | 0.057 |
|  | GeoMean | 1520 | 1770 | 1.16 | 20300 | 23800 | 1.17 |
|  | 90% CI lower | 1330 | 1550 | 1.15 | 17600 | 20700 | 1.16 |
|  | 90% CI upper | 1740 | 2020 | 1.17 | 23500 | 27300 | 1.18 |
| Fluconazole (200 mg QD) | Mean | 1930 | 3800 | 2.24 | 24400 | 57100 | 2.84 |
|  | SD | 1520 | 2170 | 0.62 | 16200 | 24400 | 1.16 |
|  | GeoMean | 1530 | 3310 | 2.17 | 19600 | 52300 | 2.67 |
|  | 90% CI lower | 1320 | 2960 | 2.05 | 16900 | 47600 | 2.48 |
|  | 90% CI upper | 1770 | 3710 | 2.29 | 22800 | 57400 | 2.87 |
| Fluconazole (400 mg QD) | Mean | 1930 | 4280 | 2.6 | 24400 | 66700 | 3.48 |
|  | SD | 1520 | 2290 | 0.857 | 16200 | 25200 | 1.70 |
|  | GeoMean | 1530 | 3790 | 2.48 | 19600 | 62200 | 3.17 |
|  | 90% CI lower | 1320 | 3400 | 2.32 | 16900 | 57300 | 2.90 |
|  | 90% CI upper | 1770 | 4220 | 2.65 | 22800 | 67600 | 3.47 |
| Erythromycin (500 mg TID) | Mean | 1820 | 2050 | 1.15 | 24000 | 27500 | 1.19 |
|  | SD | 1260 | 1320 | 0.096 | 14500 | 15200 | 0.12 |
|  | GeoMean | 1510 | 1740 | 1.15 | 20100 | 23700 | 1.18 |
|  | 90% CI lower | 1330 | 1540 | 1.13 | 17500 | 20900 | 1.15 |
|  | 90% CI upper | 1720 | 1960 | 1.17 | 23100 | 26900 | 1.20 |
| Diltiazem (120 mg BID) | Mean | 1950 | 2130 | 1.12 | 23500 | 25800 | 1.13 |
|  | SD | 1280 | 1320 | 0.070 | 14800 | 15200 | 0.080 |
|  | GeoMean | 1600 | 1790 | 1.11 | 19300 | 21700 | 1.13 |
|  | 90% CI lower | 1400 | 1570 | 1.10 | 16700 | 19000 | 1.11 |
|  | 90% CI upper | 1840 | 2040 | 1.13 | 22300 | 24800 | 1.14 |
| Fluvoxamine (100 mg BID) | Mean | 1820 | 2730 | 1.61 | 24000 | 39800 | 1.81 |
|  | SD | 1260 | 1580 | 0.402 | 14500 | 20300 | 0.683 |
|  | GeoMean | 1510 | 2380 | 1.57 | 20100 | 34900 | 1.74 |
|  | 90% CI lower | 1330 | 2130 | 1.5 | 17500 | 31000 | 1.64 |
|  | 90% CI upper | 1720 | 2660 | 1.65 | 23100 | 39300 | 1.84 |
| Quinidine (200 mg QD) | Mean | 1930 | 2320 | 1.2 | 23400 | 28100 | 1.19 |
|  | SD | 1250 | 1530 | 0.087 | 14900 | 18800 | 0.0738 |
|  | GeoMean | 1600 | 1910 | 1.19 | 19300 | 22800 | 1.19 |
|  | 90% CI lower | 1400 | 1660 | 1.18 | 16700 | 19700 | 1.17 |
|  | 90% CI upper | 1840 | 2200 | 1.21 | 22200 | 26400 | 1.20 |
| Efavirenz (600 mg QD) | Mean | 1780 | 1470 | 0.779 | 23200 | 18700 | 0.742 |
|  | SD | 1260 | 1300 | 0.231 | 13500 | 14700 | 0.262 |
|  | GeoMean | 1460 | 1070 | 0.733 | 19300 | 13100 | 0.679 |
|  | 90% CI lower | 1280 | 906 | 0.675 | 16800 | 10700 | 0.613 |
|  | 90% CI upper | 1670 | 1270 | 0.797 | 22200 | 16000 | 0.752 |
| Rifampicin (600 mg QD) | Mean | 1930 | 1010 | 0.444 | 24400 | 10100 | 0.345 |
|  | SD | 1520 | 1270 | 0.218 | 16300 | 11400 | 0.203 |
|  | GeoMean | 1530 | 591 | 0.386 | 19600 | 5610 | 0.286 |
|  | 90% CI lower | 1320 | 472 | 0.342 | 16900 | 4370 | 0.249 |
|  | 90% CI upper | 1770 | 739 | 0.437 | 22800 | 7200 | 0.329 |

AUC Ratio = (AUC_τ_ with inh)/(AUC_τ_ w/o inh); C_max_ ratio = (C_max_ with inh)/(C_max_ w/o inh); CI = confidence interval; GeoMean = geometric mean; inh = inhibition; N = the number of subjects; N/A = not available; w/o = without.

Table 11: Model-predicted PK and Drug-drug Interaction Magnitudes Following Repeated Doses (400 mg QD) of Fedratinib (as the Victim) and Repeated Doses of CYP3A4 Modulators in Cancer Patients

| Perpetrator (dosing regimen) | Parameter | C_max_ w/o inh (ng/mL) | C_max_ with inh (ng/mL) | C_max_ Ratio | AUC_τ_ w/o inh (ng/mL·h) | AUC_τ_ with inh (ng/mL·h) | AUC ratio |
| --- | --- | --- | --- | --- | --- | --- | --- |
| Ketoconazole (200 mg BID) | Mean | 3130 | 4980 | 1.67 | 50700 | 86600 | 1.85 |
|  | SD | 1730 | 2430 | 0.344 | 26700 | 37300 | 0.499 |
|  | GeoMean | 2740 | 4470 | 1.63 | 43700 | 78100 | 1.79 |
|  | 90% CI lower | 2440 | 4030 | 1.56 | 38600 | 70300 | 1.69 |
|  | 90% CI upper | 3060 | 4950 | 1.7 | 49600 | 86800 | 1.89 |
| Ketoconazole (400 mg QD) | Mean | 3130 | 4890 | 1.63 | 50700 | 83900 | 1.78 |
|  | SD | 1730 | 2430 | 0.324 | 26700 | 37100 | 0.454 |
|  | GeoMean | 2740 | 4370 | 1.6 | 43700 | 75300 | 1.72 |
|  | 90% CI lower | 2440 | 3940 | 1.53 | 38600 | 67600 | 1.63 |
|  | 90% CI upper | 3060 | 4850 | 1.67 | 49600 | 83900 | 1.82 |
| Ritonavir (100 mg BID) | Mean | 3160 | 4510 | 1.5 | 50900 | 78400 | 1.65 |
|  | SD | 1880 | 2240 | 0.235 | 28100 | 35600 | 0.358 |
|  | GeoMean | 2730 | 4040 | 1.48 | 43500 | 70200 | 1.61 |
|  | 90% CI lower | 2430 | 3640 | 1.43 | 38200 | 63000 | 1.55 |
|  | 90% CI upper | 3060 | 4470 | 1.53 | 49500 | 78200 | 1.69 |
| Itraconazole (200 mg BID at Day 1 + 200 mg QD from Day 2) | Mean | 3370 | 5480 | 1.75 | 50600 | 91100 | 2.01 |
|  | SD | 1900 | 2570 | 0.497 | 27500 | 40200 | 0.853 |
|  | GeoMean | 2920 | 4930 | 1.69 | 43400 | 82200 | 1.90 |
|  | 90% CI lower | 2590 | 4450 | 1.6 | 38100 | 74100 | 1.77 |
|  | 90% CI upper | 3290 | 5470 | 1.78 | 49300 | 91200 | 2.03 |
| Clarithromycin (250 mg BID) | Mean | 3160 | 3690 | 1.18 | 50900 | 60100 | 1.19 |
|  | SD | 1880 | 2140 | 0.056 | 28100 | 32200 | 0.061 |
|  | GeoMean | 2730 | 3200 | 1.18 | 43500 | 51800 | 1.19 |
|  | 90% CI lower | 2430 | 2860 | 1.16 | 38200 | 45800 | 1.18 |
|  | 90% CI upper | 3060 | 3590 | 1.19 | 49500 | 58700 | 1.20 |
| Fluconazole (200 mg QD) | Mean | 3260 | 5560 | 1.87 | 50000 | 95600 | 2.18 |
|  | SD | 2120 | 2800 | 0.462 | 29500 | 38500 | 0.679 |
|  | GeoMean | 2700 | 4930 | 1.82 | 42200 | 88200 | 2.09 |
|  | 90% CI lower | 2360 | 4430 | 1.74 | 37000 | 80700 | 1.97 |
|  | 90% CI upper | 3100 | 5490 | 1.91 | 48100 | 96400 | 2.22 |
| Fluconazole (400 mg QD) | Mean | 3260 | 6050 | 2.07 | 50000 | 106000 | 2.48 |
|  | SD | 2120 | 2920 | 0.596 | 29500 | 39700 | 0.897 |
|  | GeoMean | 2700 | 5420 | 2 | 42200 | 98900 | 2.34 |
|  | 90% CI lower | 2360 | 4880 | 1.9 | 37000 | 91000 | 2.18 |
|  | 90% CI upper | 3100 | 6000 | 2.12 | 48100 | 107000 | 2.52 |
| Erythromycin (500 mg TID) | Mean | 3130 | 3380 | 1.09 | 50700 | 55000 | 1.11 |
|  | SD | 1730 | 1770 | 0.061 | 26700 | 27200 | 0.083 |
|  | GeoMean | 2740 | 2990 | 1.09 | 43700 | 48300 | 1.10 |
|  | 90% CI lower | 2440 | 2680 | 1.08 | 38600 | 43000 | 1.09 |
|  | 90% CI upper | 3060 | 3330 | 1.1 | 49600 | 54300 | 1.12 |
| Diltiazem (120 mg BID) | Mean | 3310 | 3630 | 1.12 | 49500 | 55100 | 1.14 |
|  | SD | 1830 | 1910 | 0.068 | 26200 | 28000 | 0.085 |
|  | GeoMean | 2860 | 3190 | 1.11 | 42300 | 48000 | 1.13 |
|  | 90% CI lower | 2530 | 2840 | 1.1 | 37200 | 42600 | 1.12 |
|  | 90% CI upper | 3230 | 3570 | 1.13 | 48100 | 54100 | 1.15 |
| Fluvoxamine (100 mg BID) | Mean | 3130 | 4230 | 1.41 | 50700 | 72700 | 1.51 |
|  | SD | 1730 | 2070 | 0.253 | 26700 | 33400 | 0.351 |
|  | GeoMean | 2740 | 3820 | 1.39 | 43700 | 64900 | 1.48 |
|  | 90% CI lower | 2440 | 3460 | 1.35 | 38600 | 58100 | 1.42 |
|  | 90% CI upper | 3060 | 4210 | 1.44 | 49600 | 72400 | 1.54 |
| Quinidine (200 mg QD) | Mean | 3320 | 3990 | 1.2 | 49800 | 60400 | 1.21 |
|  | SD | 1880 | 2300 | 0.074 | 28400 | 35800 | 0.069 |
|  | GeoMean | 2870 | 3440 | 1.2 | 42300 | 51000 | 1.21 |
|  | 90% CI lower | 2550 | 3050 | 1.18 | 37200 | 44700 | 1.19 |
|  | 90% CI upper | 3230 | 3880 | 1.21 | 48200 | 58200 | 1.22 |
| Efavirenz (600 mg QD) | Mean | 1780 | 1470 | 0.779 | 23200 | 18700 | 0.742 |
|  | SD | 1260 | 1300 | 0.231 | 13500 | 14700 | 0.262 |
|  | GeoMean | 1460 | 1070 | 0.733 | 19300 | 13100 | 0.679 |
|  | 90% CI lower | 1280 | 906 | 0.675 | 16800 | 10700 | 0.613 |
|  | 90% CI upper | 1670 | 1270 | 0.797 | 22200 | 16000 | 0.752 |
| Rifampicin (600 mg QD) | Mean | 3270 | 2180 | 0.595 | 50200 | 27900 | 0.481 |
|  | SD | 2120 | 2070 | 0.23 | 29600 | 25600 | 0.232 |
|  | GeoMean | 2710 | 1460 | 0.539 | 42400 | 17500 | 0.413 |
|  | 90% CI lower | 2370 | 1190 | 0.484 | 37200 | 13900 | 0.361 |
|  | 90% CI upper | 3110 | 1790 | 0.6 | 48300 | 22000 | 0.471 |

AUC Ratio = (AUC_τ_ with inh)/(AUC_τ_ w/o inh); C_max_ ratio = (C_max_ with inh)/(C_max_ w/o inh); CI = confidence interval; GeoMean = geometric mean; inh = inhibition; N = the number of subjects; N/A = not available; w/o = without.

# SM10: PBPK simulations of fedratinib re-escalated after discontinuation of strong CYP3A4 inhibitors

The PBPK model of fedratinib was applied to simulate plasma fedratinib concentrations for stepwise re-escalation of fedratinib dosing after discontinuation of coadministered strong CYP3A4 inhibitors including ketoconazole and itraconazole. The simulation design and results are shown in Figure 6 in the main body and Figure 7 in SM10 for ketoconazole and itraconazole, respectively.

The simulation was performed using the default Simcyp “Cancer” population file (age: 36-83 years, female fraction: 0.5, 10 virtual trials with 6 subjects per trial) as follows.

- Fedratinib was orally administered as repeated doses to steady-state (at the recommended dose of 200 mg QD) simultaneously with ketoconazole or itraconazole for 3 weeks in “Period 1”.
- Upon discontinuation of the CYP3A4 inhibitor, the fedratinib dose was escalated by 100 mg to a total dose of 300 mg QD for 1 week (for ketoconazole) or 2 weeks (for itraconazole) in “Period 2”.
- Fedratinib dose was further escalated, 1 week (for ketoconazole) or 2 weeks (for itraconazole) after discontinuation of the CYP3A4 inhibitor, by 100 mg to the final total dose of 400 mg QD for 1 week in “Period 3”.

Figure 7: PBPK Simulation Design for Fedratinib Dose Re-escalation after Discontinuation of Itraconazole and the Simulated PK Profiles of Fedratinib in Cancer Patients


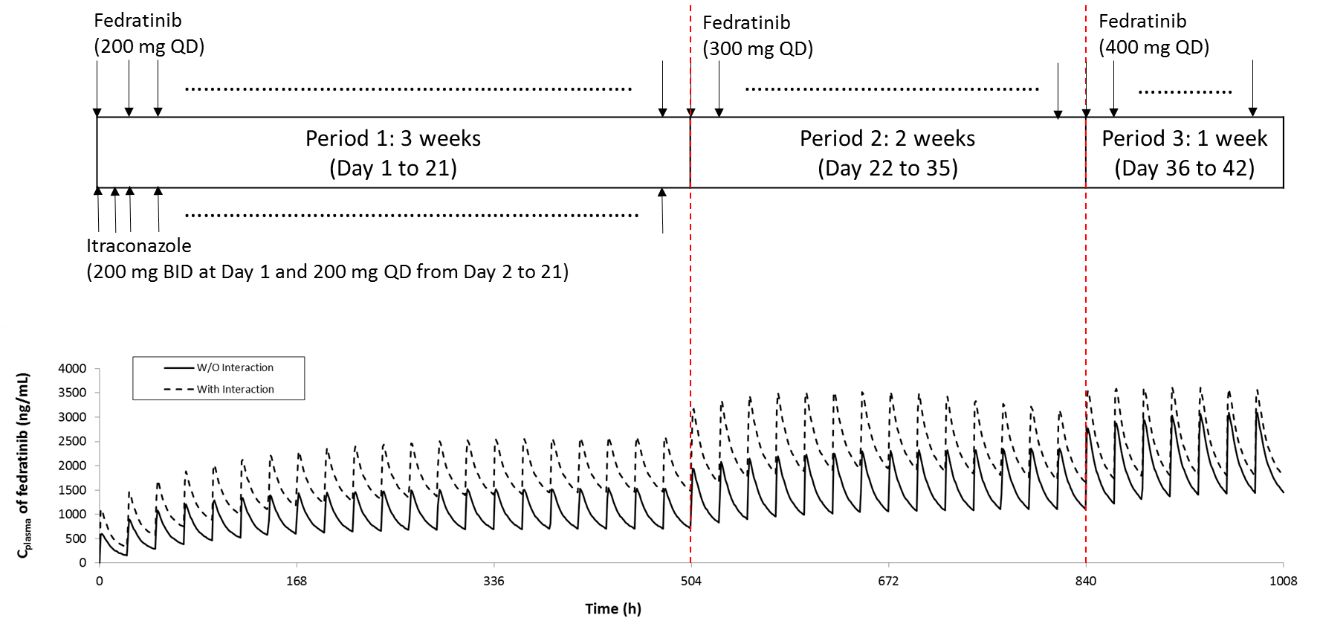


Corresponding steady-state exposure parameters were derived using non-compartment analysis (NCA) on the simulated mean concentration-time profiles in Phoenix^®^ (V7, Certara, Princeton, NJ) and are listed in Table 12. The PBPK simulations indicate that the 100-mg dose increments result in comparable PK profiles (Figure 6 in the main body and Figure 7 in SM10) and similar steady-state exposures (Table 12 in SM10) at the end of each period – forming the basis for the stepwise re-escalation recommendation.

Table 12: Summary of Fedratinib Exposure Parameters Derived from the Mean PK Profiles Simulated Under the Proposed Dose Re-Escalation Scenarios

| Relative Fedratinib AUC_τ_^a^ (%) | | |
| --- | --- | --- |
| Period 1 (Day 20) with Ketoconazole | Period 2 (Day 28) Ketoconazole discontinued | Period 3 (Day 35) Ketoconazole discontinued |
| 85 | 76 | 99 |
| Period 1 (Day 21) with Itraconazole | Period 2 (Day 35) Itraconazole discontinued | Period 3 (Day 42) Itraconazole discontinued |
| 90 | 105 | 116 |

^a^ The Relative Fedratinib AUC_τ_^a^ (%) is relative to the steady-state AUC_τ_ (i.e., 50800 ng·h /mL) predicted in cancer patients from the PBPK model for a clinical dosing regimen of 400 mg QD without a CYP3A4 inhibitor (see Table 6).

# SM 11: Derivation of transporter inhibition parameters for fedratinib

The transporter inhibition parameters (Ki) for fedratinib were derived from the experimentally determined IC50 values using the Cheng-Prusoff equation, i.e., Ki = IC_50_/(1+[S]/K_m_) [10] with the substrate concentrations ([S]) reported in the experimental studies and the substrate K_m_ values collected from literature, as summarized in Table 13.

Table 13: Converting Transporter IC50 into Ki Values for Fedratinib

| Transporter | Ki (μM) | IC_50_ (μM) | Probe substrate | [S] (μM) | K_m_ (μM) | Reference |
| --- | --- | --- | --- | --- | --- | --- |
| OCT1 | 5.59 | 6.06 | TEA | 25 | 296 | [11] |
| OCT2 | 0.71 | 0.78 | Metformin | 25 | 235 | [12] |
| BCRP | 27.8 | 29.9 | Methotrexate | 100 | 1300 | [13] |
| OATP1B1 | 14.6 | 16.4 | Estradiol-17β-glucuronide | 1 | 8.17 | [14] |
| OATP1B3 | 8.4 | 9.51 | CCK8 | 0.5 | 3.8 | [15] |
| P-gp | 10.0 | 10.1 | digoxin | 5 | 385 | [16] |

# SM 12: Estimation of impacts of distribution parameters on fedratinib PK

In the current Simcyp model, the distribution parameters were directly converted from the compartmental model parameters (see Supplemental Material SM 2 and SM3). Overall, the model captures the clinically observed mean plasma concentration-time data, while under-prediction is found at the terminal stage with time later than ~96 hours (see Figure 2A and 2B in the main body).

As a further exploration, the CLin and CLout parameters in the Simcyp® distribution module are used in replacement of the Q parameter. The CLin and CLout parameters are optimized to be 19.3 and 8.54 L/h, respectively, by minimizing the differences between the model-predicted and clinically observed mean plasma PK profiles of fedratinib in healthy subjects given single doses of 500 mg fedratinib. Following the improvement of distribution parameters while keeping the other PK parameters (including Vss, Vsac, and clearances) unchanged, the agreement between model prediction and the clinical observation at the terminal stage is significantly improved as evidenced in the log-scale plots shown below (see Supplemental Material SM 12 Figure 8 and Figure 9, in which solid curves represent simulations with the default distribution parameters and dashed curves represent simulations with the optimized distribution parameters). Subsequently, the model-predicted and clinically-observed terminal half-lives are expected to be also significantly improved for both 300 mg and 500 mg single-dose scenarios in healthy subjects. However, the impacts of the optimized distribution parameters on the model-simulated exposure parameters are minor or negligible under both single-dose and repeated-dose scenarios, as evidenced in the linear-scale plots (see Supplemental Material SM 12 Figure 8 and Figure 9) and the model-predicted exposure parameters (see SM12 Table 13).

Furthermore, it is not expected that the model applications in assessing drug-drug interaction are limited by the sub-optimal distribution parameters in the current model. For example, a further simulation shows that when the distribution parameters are changed from the current values to the optimized values, the model-predicted ketoconazole perpetrator effect on fedratinib is slightly changed from 2.09 to 1.97 in Cmax ratio and from 3.17 to 3.10 in AUC ratio, respectively.

In summary, although the model fittings at the terminal stage of the single-dose PK profiles in healthy subjects can be significantly improved by refining the distribution parameters, the current PBPK model with sub-optimal distribution parameters is (1.) adequate to predict the fedratinib PK profiles in healthy volunteers and MF patient populations, and (2.) adequate to predict the CYP3A4 inhibitor effects on fedratinib PK.

| 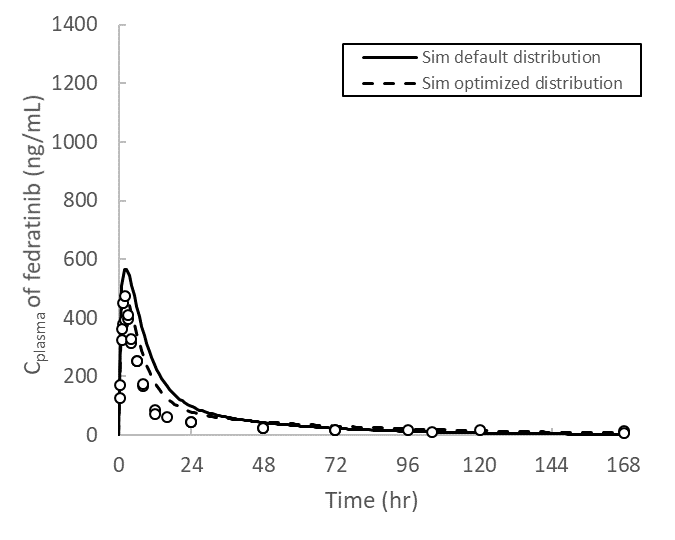  300 mg single dose (linear scale) | 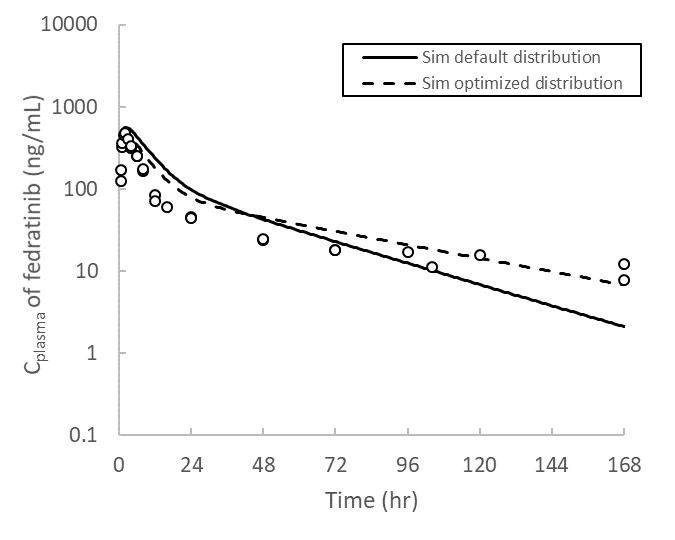  300 mg single dose (log scale) |
| --- | --- |

| 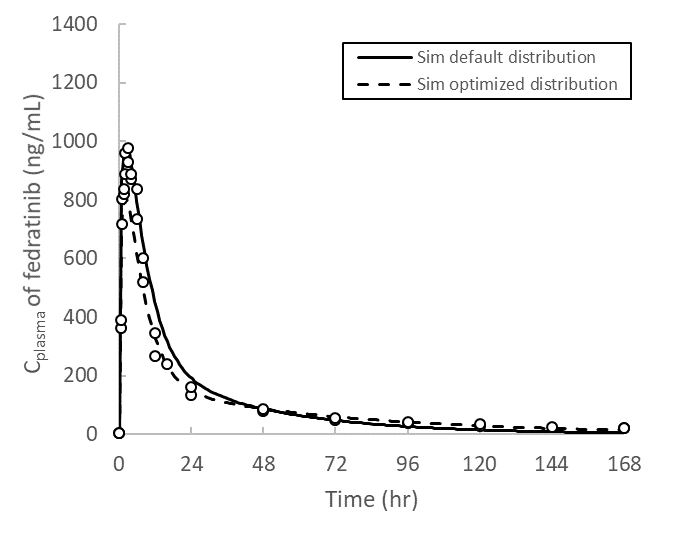  500 mg single dose (linear scale) | 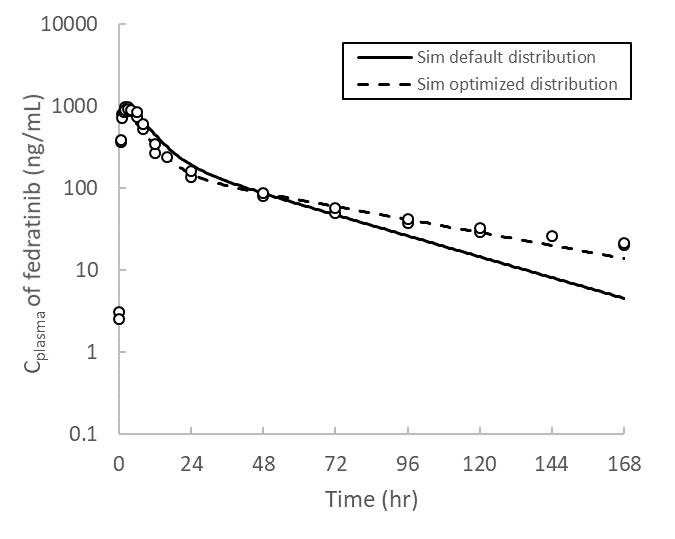  500 mg single dose (log scale) |
| --- | --- |

**Figure 8: PBPK simulations of plasma fedratinib PK profiles in healthy subjects**

| 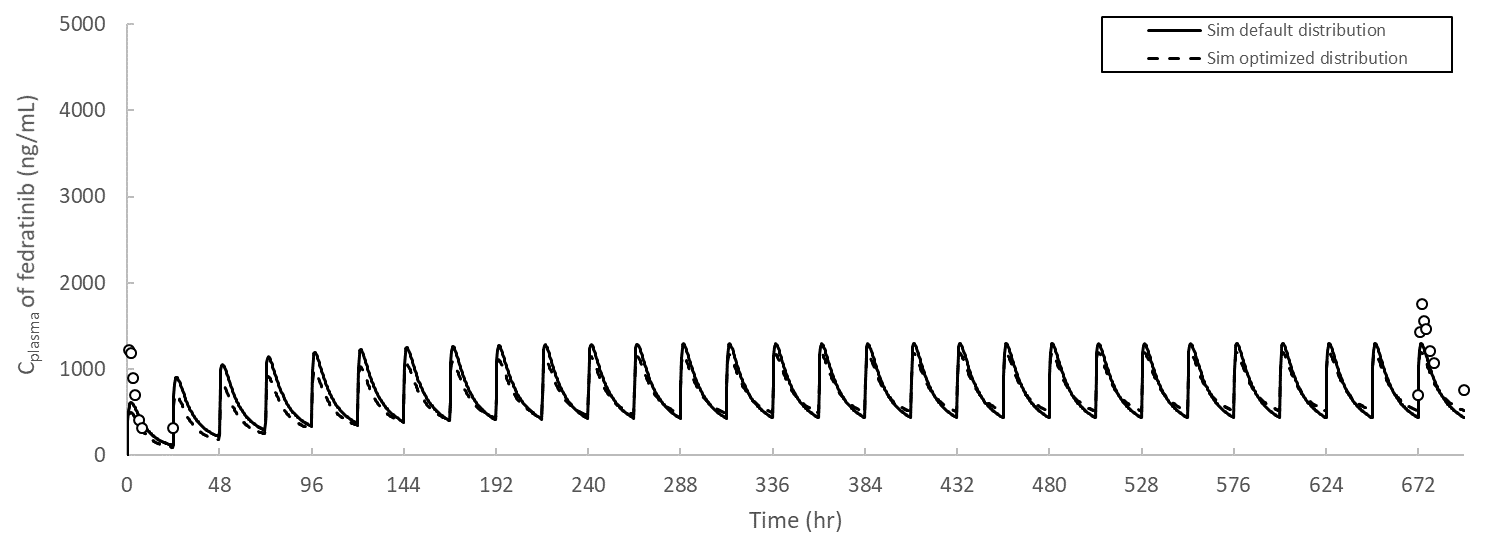  300 mg QD (linear scale) |
| --- |
| 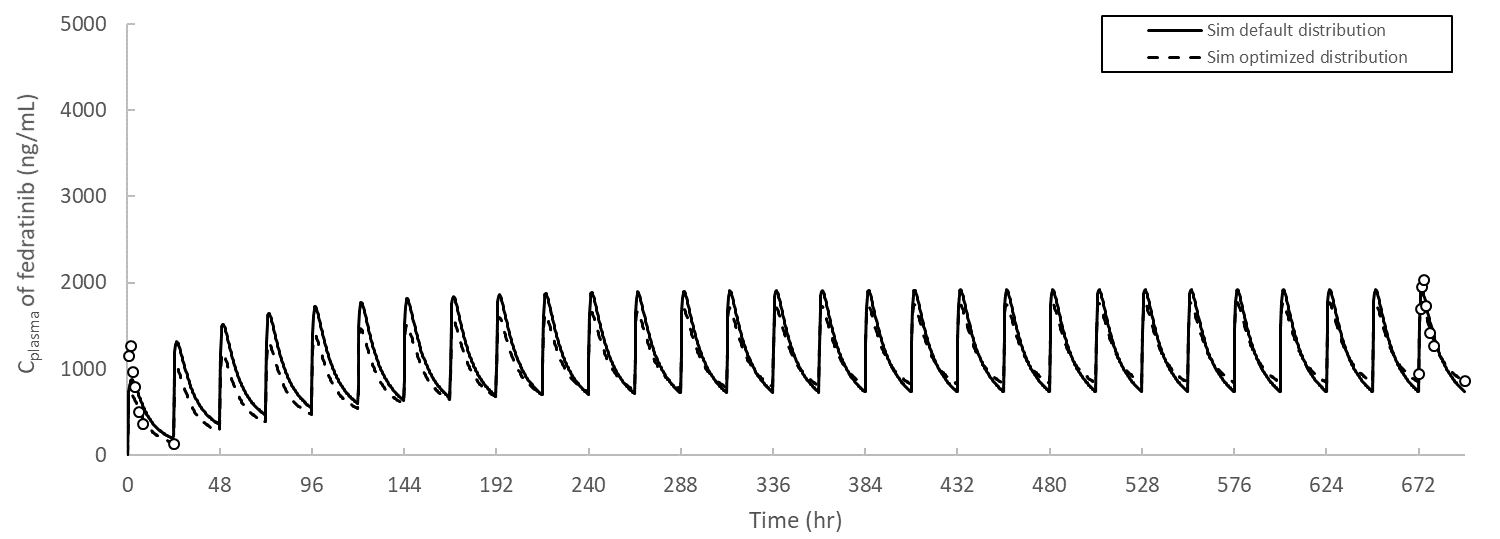  400 mg QD (linear scale) |
| 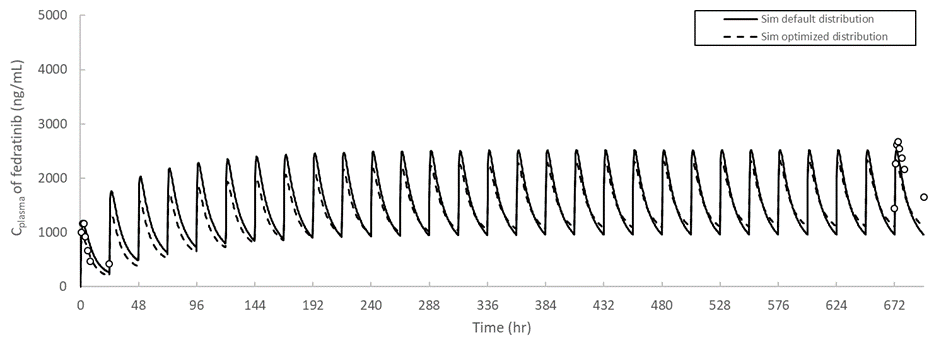  500 mg QD (linear scale) |

Figure 9: PBPK simulations of plasma fedratinib PK profiles in myelofibrosis (MF) patients

Table 13: Impacts of distribution parameters on model-predicted exposure parameters

| **Clinical Scenario** | **Distribution parameter** | **Cmax (ng/mL)** | | | **AUC (ng/mL·h)** | | |
| --- | --- | --- | --- | --- | --- | --- | --- |
|  |  | **Observation** | **Prediction** | **PE (%)** | **Observation** | **Prediction** | **PE (%)** |
| 300 mg single dose in healthy subjects | Default | 497 | 551 | 11 | 6530 | 8560 | 31 |
|  | Optimal |  | 465 | -6.4 |  | 8290 | 21 |
| 500 mg single dose in healthy subjects | Default | 683 | 950 | 39 | 13200 | 16100 | 22 |
|  | Optimal |  | 796 | 17 |  | 15400 | 17 |
| 300 mg repeated doses in MF patients (Day 1) | Default | 1070 | 584 | -45 | 7020 | 6530 | -7 |
|  | Optimal |  | 485 | -55 |  | 5190 | -26 |
| 300 mg repeated doses in MF patients (Steady state) | Default | 1530 | 1240 | -19 | 22400 | 15900 | -29 |
|  | Optimal |  | 1150 | -25 |  | 15900 | -29 |
| 400 mg repeated doses in MF patients (Day 1) | Default | 1290 | 834 | -35 | 9130 | 9760 | 7 |
|  | Optimal |  | 689 | -47 |  | 7640 | -16 |
| 400 mg repeated doses in MF patients (Steady state) | Default | 1800 | 1800 | 0 | 26900 | 24200 | -10 |
|  | Optimal |  | 1680 | -6.7 |  | 24200 | -10 |
| 500 mg repeated doses in MF patients (Day 1) | Default | 1270 | 1100 | -13 | 9980 | 13300 | 33 |
|  | Optimal |  | 898 | -29 |  | 10200 | 2 |
| 500 mg repeated doses in MF patients (Steady state) | Default | 2540 | 2440 | -4 | 38700 | 33200 | -14 |
|  | Optimal |  | 2260 | -11 |  | 33200 | -14 |

AUC refers to AUC_inf_ for the single dose scenarios and AUCτ for the repeated QD dose scenarios. The number of subjects (n) = 6 in TDU12620, n = 17 in INT12894, n = 10-11 in ARD11936, and n = 100 in virtual trial simulations, respectively. All model simulations listed in the table were simulated using the default “Healthy Volunteers” population files provided in Simcyp® (V17R1). The prediction error is calculated using PE = (GM_prediction_ – GM_observation_)/GM_observation_ ×100%, where GM refers to geometric mean. All values listed in the table refer to GM values.

# References

1. Zhang, M., et al., *Effect of food on the bioavailability and tolerability of the JAK2-selective inhibitor fedratinib (SAR302503): Results from two phase I studies in healthy volunteers.* Clin Pharmacol Drug Dev, 2015. **4**(4): p. 315-21.

2. Ogasawara, K., et al., *Population pharmacokinetics of fedratinib in patients with myelofibrosis, polycythemia vera, and essential thrombocythemia.* Cancer Chemother Pharmacol, 2019. **84**(4): p. 891-898.

3. FDA, *NDA212327: Cross-Discipline Team Leader Review*. 2019.

4. NIH, *Drug Interaction Study of SAR302503 in Patients With Solid Tumor (ClinicalTrials.gov Identifier: NCT01585623)*.

5. Ogasawara, K., et al., *Effects of repeated oral doses of ketoconazole on a sequential ascending single oral dose of fedratinib in healthy subjects.* Cancer Chemother Pharmacol, 2020.

6. Zhang, M., et al., *A randomized, placebo-controlled study of the pharmacokinetics, pharmacodynamics, and tolerability of the oral JAK2 inhibitor fedratinib (SAR302503) in healthy volunteers.* J Clin Pharmacol, 2014. **54**(4): p. 415-21.

7. FDA, *INREBIC® (fedratinib) capsules, for oral use (USA drug label)*. 2019.

8. Amin, M.L., *P-glycoprotein Inhibition for Optimal Drug Delivery.* Drug Target Insights, 2013. **7**: p. 27-34.

9. Almond, L.M., et al., *Prediction of Drug-Drug Interactions Arising from CYP3A induction Using a Physiologically Based Dynamic Model.* Drug Metab Dispos, 2016. **44**(6): p. 821-32.

10. Cheng, Y. and W.H. Prusoff, *Relationship between the inhibition constant (K1) and the concentration of inhibitor which causes 50 per cent inhibition (I50) of an enzymatic reaction.* Biochem Pharmacol, 1973. **22**(23): p. 3099-108.

11. Mulgaonkar, A., et al., *Human organic cation transporters 1 (SLC22A1), 2 (SLC22A2), and 3 (SLC22A3) as disposition pathways for fluoroquinolone antimicrobials.* Antimicrob Agents Chemother, 2013. **57**(6): p. 2705-11.

12. Sogame, Y., et al., *Transport of biguanides by human organic cation transporter OCT2.* Biomed Pharmacother, 2013. **67**(5): p. 425-30.

13. Breedveld, P., et al., *The effect of low pH on breast cancer resistance protein (ABCG2)-mediated transport of methotrexate, 7-hydroxymethotrexate, methotrexate diglutamate, folic acid, mitoxantrone, topotecan, and resveratrol in in vitro drug transport models.* Mol Pharmacol, 2007. **71**(1): p. 240-9.

14. Izumi, S., et al., *Substrate-dependent inhibition of organic anion transporting polypeptide 1B1: comparative analysis with prototypical probe substrates estradiol-17beta-glucuronide, estrone-3-sulfate, and sulfobromophthalein.* Drug Metab Dispos, 2013. **41**(10): p. 1859-66.

15. Hirano, M., et al., *Contribution of OATP2 (OATP1B1) and OATP8 (OATP1B3) to the hepatic uptake of pitavastatin in humans.* J Pharmacol Exp Ther, 2004. **311**(1): p. 139-46.

16. Hansen, T.S. and O.G. Nilsen, *Echinacea purpurea and P-glycoprotein drug transport in Caco-2 cells.* Phytother Res, 2009. **23**(1): p. 86-91.
